# Secretome Analysis of Prostate Cancer Cell Lines Reveals Cell Cycle-Dependent PSA Secretion and Potential Biomarkers

**DOI:** 10.3390/cancers17050721

**Published:** 2025-02-20

**Authors:** Eshwari Dathathri, Yvette Peters, Diana Andreoli, Mel Bruins, Jaco Kraan, Leon W. M. M. Terstappen, Ruchi Bansal

**Affiliations:** 1 Department of Medical Cell Biophysics, Technical Medical Center, Faculty of Science and Technology, University of Twente, 7522 NB Enschede, The Netherlands; e.dathathri@utwente.nl (E.D.); yvette.peterss@gmail.com (Y.P.); d.s.andreoli@utwente.nl (D.A.); melbruins@ziggo.nl (M.B.); r.bansal@utwente.nl (R.B.); 2 Department of Medical Oncology, Erasmus MC Cancer Institute, University Medical Center, 3015 GD Rotterdam, The Netherlands; j.kraan@erasmusmc.nl; 3 Department of General, Visceral and Pediatric Surgery, University Hospital Düsseldorf, Heinrich-Heine University, 40225 Düsseldorf, Germany

**Keywords:** prostate-specific antigen (PSA), single-cell secretion, anti-androgen, castration-naïve prostate cancer, castration-resistant prostate cancer, cell cycle, progranulin, cathepsin D

## Abstract

**Background:** Metastatic prostate cancer (mPCa) is marked by heterogeneity and therapy resistance, which arise from prolonged therapy regimens. This heterogeneity is reflected in various morphologic and genetic characteristics, biomarker expression, and other molecular mechanisms, thereby contributing to the complexity of the disease. **Methods:** To investigate tumor heterogeneity, the effects of androgen targeting therapy (ADT) on single-cell PSA secretion was assessed by analyzing the prostate cancer cell lines using a modified ELISpot platform. The FACS and cytospin techniques were employed to understand the influence of the cell cycle on PSA secretion patterns. Additionally, a proteome array was used to identify potential biomarkers from different PCa cell lines with varying metastatic potential. **Results:** Among the various PCa cell lines examined, PSA expression and secretion could be visualized only from the LNCaPs. PSA secretion from circulating tumor cells (CTCs) further confirmed the validity of this assay. These LNCaPs exhibited heterogeneity in single-cell intracellular and extracellular PSA expression and in their ADT responses. LNCaPs in the G1 phase showed higher PSA secretion than in the S or G2/M phase. Apart from PSA, Cathepsin D, Progranulin, IL-8, Serpin E1, and Enolase 2 were identified as secretome markers from the metastatic PCa cell lines. **Conclusions:** We observed variability in PSA secretion in LNCaP in response to anti-androgen treatment and a cell cycle-dependent secretion pattern. The notable presence of Progranulin and Cathepsin D in metastatic cell lines makes them promising candidates for use in multiplexing and single-cell platforms, potentially advancing our understanding and treatment of this disease.

## Simple Summary

Metastatic prostate cancer (mPCa) is a complex disease, characterized by tumor heterogeneity and resistance to therapy. This study explores PSA secretion from single cells from various PCa cell lines to investigate tumor heterogeneity under the influence of anti-androgen therapy. In LNCaP cells, PSA secretion was found to be cell cycle-dependent. To improve the assessment of cell secretion in response to treatment, Progranulin and Cathepsin D were identified as promising secretome markers in addition to PSA, from metastatic PCa cell lines.

## 1. Introduction

Prostate cancer (PCa) is one of the most common malignancies affecting the male population and is the second leading cause of cancer-related death in the Western world [1]. While many cases of PCa are characterized by slow progression and low risk, a subset exhibits aggressive behavior, leading to metastatic castration-naïve prostate cancer (CNPC) and finally progressing to the lethal stage of metastatic castration-resistant prostate cancer (CRPC) [2].

Localized PCa is usually treated with surgery or radiotherapy with a 5-year survival rate of nearly 100%, while for metastatic cancer, it is only 30%. With androgen deprivation therapy (ADT), using androgen receptor signaling inhibitors (ARSIs) such as Enzalutamide, Abiraterone, and Apalutmide improves overall survival in patients with Androgen Receptor (AR)-sensitive tumors. AR treatment inevitably leads to the development of castration-resistant prostate cancer (CRPC) and tumor heterogeneity. To address this, treatments targeting different tumor mechanisms such as DNA repair, tumor plasticity, and cell cycle abnormalities will be required to overcome resistance [3].

Tumor heterogeneity poses a unique challenge in making treatment decisions, and the lack of reliable biomarkers that can predict therapeutic efficacy represents a significant gap in PCa management. In this context, liquid biopsy has emerged as a useful approach, offering minimally invasive means to detect alterations in the proteome and genome by characterizing circulating tumor cells (CTCs), tumor-derived extracellular vesicles (EVs), circulating proteins, and circulating nucleic acids. This characterization can provide insights into the composition of the tumor, its heterogeneity, real-time changes in the composition during disease progression, and the effects of treatment [4]. Although EVs and circulating nucleic acids are more abundant in patient blood, CTCs contain all the information and are thus preferred for investigation of tumor composition [5]. The rarity of CTCs however prevents tumor characterization in most cancer patients. This can be overcome through Diagnostic Leukapheresis (DLA) [6,7].

Assuming that enough CTCs can be harvested in metastatic PCa patients, in this study, we focused on the feasibility of studying PCa secretome using various PCa cell lines representing different PCa disease stages (benign, hormone-sensitive, and castration-resistant). We first explored the secretion of prostate-specific antigen (PSA) from single cells in response to ADT such as Enzalutamide, using the modified ELISpot platform. To validate this assay, we measured PSA secretion from CTCs obtained from the DLA of two CNPC patients. Secondly, we evaluated the influence of the cell cycle on secretion patterns to improve our understanding of tumor biology. Lastly, we investigated other proteins that may complement PSA as potential biomarkers for PCa.

## 2. Materials and Methods

### 2.1. Cell Lines and Culturing

The PCa cell lines PWR-1E (RRID: CVCL_3775), RWPE-1 (RRID: CVCL_3791), RWPE-2 (RRID: CVCL_3792), LNCaP (RRID: CVCL_0395), 22Rv1 (RRID: CVCL_1045), and PC3 (RRID: CVCL_0035) were obtained from the American Tissue Culture Collection (ATCC, Manassas, VA, USA). An overview of the properties of the cell lines are provided in Supplementary Table S1. The hepatocellular carcinoma cell line, HepG2 (RRID: CVCL_0027), used as a control cell line, was obtained from the American Tissue Culture Collection (ATCC). All cell lines used in this study were regularly tested for the absence of mycoplasma contamination using standard PCR assays and were authenticated by short tandem repeat (STR) analysis.

All cells were cultured in T25-treated culture flasks (VWR International B.V., Amsterdam, The Netherlands) at 37 °C in an atmosphere containing 5% CO_2_ and harvested when they reached 70–80% confluency. The cells of RWPE-1, RWPE-2, and PWR-E1 were cultured in Keratinocyte serum-free medium (SFM) supplemented with human epidermal growth factor (EGF) and bovine pituitary extract (BPE) also obtained from ATCC. To subculture the RWPE-1, RWPE-2, and PWR-1E cells, cells were first washed with Dulbecco’s phosphate-buffered saline (DPBS) (Lonza, Verviers, Belgium). The cells were trypsinized with a 1:1 dilution of 0.05% trypsin-EDTA (Gibco, ThermoFisher Scientific, Waltham, MA, USA) in DPBS and incubated for at least two minutes. Cells were collected in DPBS + 2% Fetal Bovine Serum (FBS) (Sigma Aldrich, St. Louis, MO, USA) to deactivate the trypsin. The desired cell concentration was added to a new tissue culture flask with a fresh cell culture medium. All three cell lines were seeded with a seeding density of 5000 cells/cm^2^.

LNCaP, PC3, and 22Rv1 were cultured in Roswell Park Memorial Institute (RPMI 1640) supplemented with L-glutamine (Lonza), 10% Fetal Bovine Serum (FBS) (Sigma Aldrich) and 100 U/mL Penicillin and 100 µg/mL Streptomycin (Lonza). The cells were washed with phosphate-buffered saline (PBS) (Sigma Aldrich). Prewarmed 0.05% trypsin-EDTA was added and incubated for 1 min at 37 °C. When cells were detached, the cells were collected in fresh medium with pipetting and counted using the Luna-FLTM automated cell counter (Westburg B.V., Leusden, The Netherlands). The cells were subcultured into new flasks and maintained in a humidified incubator at 37 °C and 5% CO_2_. LNCaP cells were passaged with a seeding density of 5000 cells/cm2, 22Rv1 with a seeding density of 10.000 cells/cm^2^, and PC3 with 5.000 cells/cm^2^.

HepG2 cells were cultured in Dulbecco’s modified Eagle’s medium (DMEM) (Gibco) supplemented with 10% FBS and 1% penicillin/streptomycin washed with PBS before adding 0.05% trypsin-EDTA and incubating for 5 min. Cell culture medium was added to neutralize the trypsin. With a seeding density of 5000 cells/cm^2^, the cells were passaged to a new tissue culture flask with fresh cell culture medium.

### 2.2. Patient Samples

PSA secretion was measured using the circulating tumor cells (CTCs) obtained from the diagnostic leukapheresis (DLA) samples of 2 metastatic castration-naïve prostate cancer (CNPC) patients before the initiation of androgen deprivation therapy (ADT). Both patients participated in the PICTURES (Trial ID: NL8549) study in which patients were screened for the presence of CTC and underwent a DLA procedure when the CellSearch CTC count in 7.5 mL of blood was 3 or higher. This study was performed in agreement with the Helsinki Declaration, and the protocol of the PICTURES study (MEC 20-0422) was approved by the involved Medical Research Ethics Committees.

### 2.3. Effect of Androgen Inhibitors on Intercellular PSA in Cell Lines

#### 2.3.1. Cell Line Seeding and Treatment

One flask of each cell line was stimulated with R1881 [0.5 nM] (Biotang Inc., Lexington, MA, USA) overnight. A second flask for each cell line was kept untreated overnight to maintain comparable confluency. After 24 h, the cells from the second flask were trypsinized and split into two halves. One half was treated with R1881 [0.5 nM] and 100,000 cells were seeded in a 24-well plate (VWR international B.V.) The second half was left untreated, and 100,000 cells were seeded in a 24-well plate. The stimulated cells from the first flask were trypsinized and seeded in the wells plate. Enzalutamide [2 µM] (MDV3100, Selleck hem, Houston, TX, USA) was added to the wells with R1881-treated cells (first flask).

The wells plate was incubated for 24 h, and the same procedure was repeated for 48 h at 37 °C and 5% CO_2_ (as indicated in Supplementary Figure S1). Subsequently, the cells were fixed with 1% formalin (Sigma Aldrich). The cells were permeabilized by washing with 0.1% Triton X-100 (Sigma Aldrich) in PBS for 15 min. Next, the cells were washed twice with PBS and after that 1% Bovine Serum Albumin (BSA) (Sigma Aldrich) in PBS was added for 30 min.

#### 2.3.2. Immunofluorescence Staining and Imaging

For staining, a primary antibody solution (Rabbit to PSA Ab, Cat. No.19554, Abcam, Cambridge, UK) was prepared [2 µg/mL] in 0.1% BSA in PBS and incubated for 1 h at room temperature. The wells were gently washed with PBS. A secondary antibody solution was prepared [2 µg/mL] (Goat pAb to Rb IgG, Alexa Fluor 488, Cat. No. 150077, Abcam) in 0.1% BSA in PBS in combination with PSMA-PE antibody (FOLH1) [1 µg/mL] (Cat. No. 342504, BioLegend, San Diego, CA, USA) and the cells were incubated for 1 h at room temperature.

After incubation, the wells were washed gently with PBS; DAPI [4 µM] (CellSearch, Menarini Silicon Biosystems, Bologna, Italy) was added to the wells and incubated for 15 min. The wells were washed with PBS and stored in PBS at 4 °C. With a fluorescence microscope (Nikon, Eclipse Ti, Minato, Japan), the cells were imaged. In the DAPI channel, an exposure time of 50 milliseconds (ms) was used, and in the FITC and PE channels, an exposure time of 200 ms was used.

### 2.4. Effect of Androgen Stimulation and Inhibition on PSA Secretion from Single Cells

#### 2.4.1. Preparation of PVDF Membranes

Under sterile conditions, PVDF membranes (Immuno-Blot, 0.45 µm pore size) (Bio-Rad Laboratories B.V., Veenendaal, the Netherlands) were placed in a 24-wells plate and incubated with methanol (100%) for 1 min to activate them. Membranes were washed twice with PBS. Subsequently, a monoclonal mouse anti-PSA antibody solution (Cat. No. 10-P21A, Fitzgerald Industries International, Acton, MA, USA) with a concentration of 25 µg/mL in PBS was prepared and added to the membranes (Supplementary Figure S2, step 1). The membranes were incubated overnight at 4 °C. Following the incubation, the antibody solution was removed and a 3% BSA in PBS solution was added to block the membranes for 1 h. Membranes were finally washed with PBS and placed in a fresh 24-well plate.

#### 2.4.2. Capturing PSA from Cells on PVDF Membranes Using the EliSPOT Method

Three tubes containing cell suspensions of untreated, R1881 stimulated, and Enzalutamide inhibited cells were prepared for cell lines PWR-1E, RWPE-1, RWPE-2, LNCaP, 22Rv1, PC3 and HepG2 as explained in Section 2.3.1 and illustrated in Supplementary Figure S1. 2000 cells from these suspensions were added on the membranes (prepared as per Section 2.4.1) placed in the 24 wells plate and incubated at 37 °C and 5% CO_2_ (Supplementary Figure S2, step 2). After the first 24 h, the cell culture medium was removed. These membranes were washed with 1% Tween 20 (Sigma Aldrich) in PBS for 30 min on the Thermoshaker (Thermomixer C, Eppendorf, Hamburg, Germany) at 300 rpm to remove membrane-bound cells (Step 3 of Supplementary Figure S2), followed by a final washing step with 1% BSA in PBS for 30 min This procedure was repeated for the cells and membranes after a 48 h timepoint.

#### 2.4.3. Detection of PSA Spots

To visualize the PSA, which is captured on the membranes, a primary antibody [2 µg/mL] (Rabbit anti-PSA antibody, Cat. No. 19554, Abcam) was diluted in 1% BSA in PBS. The membranes were incubated with the antibody solution and placed on the Thermoshaker for 1 h at 300 rpm. The membranes were washed three times for 5 min with 1% BSA in PBS. Subsequently, a secondary antibody was prepared [2 µg/mL] (polyclonal goat anti-Rabbit antibody, Alexa Fluor 488, Cat. No.150077, Abcam) in 1% BSA in PBS, added to the membranes and placed on the Thermoshaker for 1 h at 300 rpm Supplementary Figure S2, step 4). Following this, the membranes were washed three times for 5 min with 1% BSA in PBS. The stained membranes were dried and placed between two microscope slides. Using the VyCAP scanning program, the membranes were imaged using an inverted microscope (Nikon, Eclipse Ti) in the FITC channel using an exposure time of 100 ms, with a 10x objective (Supplementary Figure S2, step 5). The list of antibodies used for the staining of membranes is listed in Supplementary Table S2.

#### 2.4.4. Quantification of PSA Spots

The quantification was performed using the Image J software (v. 1.54g). A montage of the individual images was made and saved as a TIFF file. The image was duplicated, and the duplicated image was saved as an 8-bit image. Then, a threshold was set on the duplicated image with a minimum pixel intensity of 5500. A binary image was created and linked to the original image. The area and the mean intensity of the regions of interest were computed. The values of the mean intensity were normalized to the membranes of the HepG2 cells. The relative PSA secretions were calculated by multiplying the normalized mean intensity of the spots and the corresponding spot areas.

### 2.5. PSA Secretion Analysis Using ELISA

To detect and quantify the concentration of PSA secreted by the PCa cell lines in response to stimulation and inhibition, an ELISA was performed using the antibodies listed in Supplementary Table S2. 100,000 cells were seeded in a 24-well plate. Half of the cells were untreated (Control), and the other half were androgen stimulated using R1881. The culture medium was collected after 48 h to perform the ELISA.

A monoclonal mouse anti-PSA antibody solution [25 µg/mL] (Cat. No. 10-P21A, Fitzgerald Industries International) in PBS was prepared and used to coat a 96 microwell immune plate (172164, Sigma Aldrich) and incubated overnight. The next day, the wells were washed with 1% Tween 20 (Sigma Aldrich) and 1% BSA in PBS was incubated for 1 h. The culture medium derived from RWPE-1, RWPE-2, PWR-1E, 22Rv1, and PC3 cells were diluted in 1% BSA and 5% FBS in PBS, and samples for a calibration curve were prepared. The culture medium derived from LNCaP cells was diluted 400 times. For the calibration curve, serial dilutions of PSA (Cat. No. ab41421, Abcam, Cambridge, UK) were made from 0 to 60 ng/mL. The wells were washed again and the diluted samples and the samples for the calibration curve were incubated (100 µL/well) for 2 h. Next, a primary antibody [2 µg/mL] (Rabbit anti-PSA antibody, Cat. No.19554, Abcam) was prepared in 1% BSA in PBS and incubated for 1 h. The wells were washed again and an anti-rabbit-HRP [3 µg/mL] (Dako, Agilent, Santa Clara, USA) solution in 1% BSA in PBS was prepared and incubated for 20 min in the dark. The wells were washed again and streptavidin (Lot. P300867, R&D systems, Minneapolis, MN, USA) diluted in 1% BSA in PBS [dilution1:40] was added. After washing 1-Step™ Ultra TMB-ELISA Substrate Solution (ThermoFisher Scientific) was incubated until a clear difference in different shades of blue was visible in the standard. Subsequently, 1.8 M H_2_SO_4_ (Sigma Aldrich) in MilliQ was prepared and added to the wells. The absorbance was measured using a VictorX3 microplate reader (PerkinElmer, Waltham, MA, USA).

### 2.6. DLA Procedure

The DLA procedure was performed at the Department of Hematology at the Erasmus Medical Center using the Spectra Optia Cell Separator machine (Terumo BCT, Lakewood, CO, USA). A maximum volume of 6 L of peripheral circulating blood was processed, and citrate dextrose solution A was used as an anticoagulant.

### 2.7. CTC Enrichment from DLA and PSA Capture on PVDF Membranes

A portion of the DLA products ranging from 2 × 10^8^ to 20 × 10^8^ WBCs were shipped to the Medical Cell BioPhysics (MCBP) labs of the University of Twente. The DLA samples were processed immediately upon arrival (within 26 h after leukapheresis). DLA aliquots containing 2 × 10^8^ WBCs were transferred to a conical tube, supplemented with the dilution buffer (total volume 11 mL). The samples were then processed with the CellSearch CTC Profile kit (Menarini, Bologna IT) and immunomagnetically enriched with EpCAM ferrofluids. PVDF membranes were coated and prepared for cell seeding as described in Section 2.4.1. A DLA volume equivalent to 1 × 10^8^ WBCs was added to the membranes and incubated for 24 h to collect PSA secretion as per Section 2.4.2 and subsequent visualization of PSA spots was performed as per Section 2.4.3.

### 2.8. Effect of Cell Cycle on PSA Secretion

Since LNCaP cells showed the greatest PSA secretion and response to treatment, we explored the effect of cell cycle phases on PSA secretion by LNCaP cells.

#### 2.8.1. Cell Preparation and Staining

To analyze different cell cycle phases based on the DNA content, LNCaP cells were first harvested from culture when they reached approximately 80% confluency. The cells were then centrifuged at 300× *g* for 5 min and were resuspended in a solution of 2% FBS in PBS and a concentration of 300,000 cells per 200 µL in each FACS tube was prepared. Hoechst 33342 was added to each sample at a concentration (100 ug/mL) of 1 µL per 100 µL of cell suspension. The cells were incubated with the dye for 30 min on ice to allow adequate staining while minimizing cell stress and dye toxicity. After staining, the cells were washed by adding 300 µL of 2% FBS in PBS to bring the total volume to 500 µL. The cells were then centrifuged to remove excess dye and were resuspended in 200 µL 2% FBS in PBS ready to be FACS sorted as indicated in Supplementary Figure S3, step 1.

#### 2.8.2. FACS and Analysis

The stained cells were analyzed using Fluorescence-Activated Cell Sorting (FACS), where they were categorized according to their cell cycle phase. The cells were sorted into three groups: G1, S, and G2/M phases. The flow cytometer was set up and calibrated for UV excitation (340 to 380 nm) and detection of Hoechst 33342 fluorescence at blue wavelengths. Adjustments were made to accurately gate single cells and exclude doublets using pulse-width/pulse-area analysis. A negative control (non-stained cells) was used to set the baseline fluorescence and adjust the sensitivity of the flow cytometer. The stained cells were analyzed by flow cytometry. Hoechst 33342 fluorescence was measured to determine the DNA content of the cells, allowing the identification of cells in G1, S, and G2/M phases. Cells were sorted based on their DNA content into different collection tubes for cytospin and seeding on PVDF membranes.

#### 2.8.3. Cytospin and Visualization

After sorting, the cells were counted, and 10,000 cells from each cell cycle phase group were selected for cytospin analysis as indicated in step 2 of Supplementary Figure S3. The selected cells were spun down onto coverslips using the cytospin technique. This involved placing the cells into a specialized chamber, where they were centrifuged, spreading them evenly across the surface of the coverslip. The coverslips with spun-down cells were mounted onto microscope slides using the antifade mounting solution, preserving the fluorescent signal for extended analysis. The slides were then examined under a fluorescence microscope using the DAPI filter to visualize the Hoechst-stained nuclei. This allowed for a detailed examination of each cell cycle phase and a comparison of cellular characteristics between the different phases.

#### 2.8.4. PSA Secretion from Sorted Cells in Different Cell Cycle Phases

To investigate the secretion dynamics of Prostate-Specific Antigen (PSA) across different cell cycle phases, the secretions from the sorted cells were captured and visualized using the PVDF membranes as indicated in step 3 of Supplementary Figure S3. The membranes were prepared as mentioned in Section 2.4.1. Cells sorted into different cell cycle phases (G1, S, and G2/M) using FACS were seeded onto the prepared PVDF membranes. The cells on the membranes were incubated overnight and washed afterward as per the protocol explained in Section 2.4.2. To visualize the PSA spots, the membranes were stained and imaged as mentioned above in Section 2.4.3.

### 2.9. Proteome Profiling Array

To identify other potential biomarkers apart from PSA, a proteome profiling array was utilized, specifically the Proteome Profiler™ Array Human XL Oncology Array Kit (Cat. No. ARY026). The cells of LNCaP, PC3, and 22Rv1 cell lines were initially seeded in 6-well plates at a density of 1 × 10^6^ cells per well and cultured for two days. After this period, 2 mL of culture medium from each well was carefully collected. This duration was chosen to allow for adequate protein secretion into the medium, ensuring a robust analysis. The collected culture medium derived from different cell lines was then processed as per the standard Proteome array protocol. To control for proteins inherently present in the medium, a control sample consisting only of the culture medium was analyzed concurrently. The pixel intensities of the resulting spots were extracted from the array images. The values were normalized against a negative control to correct for background noise and variability in staining intensity. Next, a threshold was set to differentiate between true signal and background noise. The resulting true signal was acquired for the proteins from the cell lines of LNCaP, PC3, and 22Rv1.

### 2.10. Dot Blot Assay

The culture medium of LNCaP, PC3, 22Rv1, RWPE-1, and HepG2 cells was collected from culture flasks when the cells reached 70–80% confluency. A volume of 2 μL of the culture medium derived from the cell lines and a 1:10 dilution of the same were spotted on the nitrocellulose membranes and incubated for 10 min. After incubation, the membranes were blocked with 3% BSA (Sigma Aldrich) for 30 min. Following this, the membranes were incubated with the primary antibodies for an hour, followed by washing three times for 5 min. Next, staining with secondary antibodies with incubation for an hour, followed by washing three times for 5 min. The details of the antibodies used for Progranulin, STEAP2, Prostein, Cathepsin D, and GAPDH are provided in Supplementary Table S2. The membranes were dried and imaged with the inverted microscope (using Texas red filter) for an exposure time of 30 ms.

### 2.11. Statistical Analysis

The graphs were created using software GraphPad Prism (Version 5, GraphPad Software, San Diego, CA, USA). Differences between two conditions were assessed using the Mann–Whitney U test followed by the Bonferroni- Dunn post hoc test. For comparisons involving three conditions, the Kruskal–Wallis test was used, followed by Dunn’s post hoc test for pairwise comparisons. The covariates are presented as the median (IQR) for each group. Differences with *p*-values < 0.05 were considered statistically significant and denoted as follows: * *p* < 0.05, ** *p* < 0.01, *** *p* < 0.001, and **** *p* < 0.0001.

## 3. Results

### 3.1. Androgen Treatment Influences PSA Expression and Growth of LNCaPs

**Figure 1:**
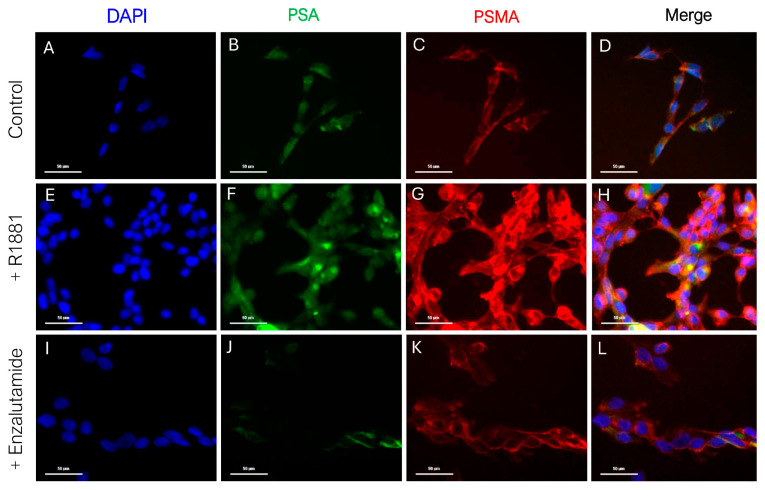
Immunofluorescence staining of LNCaP cells. The control cells are stained with DAPI for nuclear visualization in blue (**A**), PSA (FITC) in green (**B**), and PSMA(PE) in red (**C**). The R1881-stimulated cells are stained similarly with DAPI (**E**), PSA (**F**), and PSMA (**G**). The enzalutamide-inhibited cells are stained with DAPI (**I**), PSA (**J**), and PSMA (**K**). The overlay of all channels for the control, R1881-stimulated and enzalutamide-inhibited cells are shown in (**D**), (**H**) and (**L**) respectively. Scale bar = 50 μm.

Treatment with ADT often encounters resistance, as patients with heterogeneous tumors respond differently when exposed to therapy. To obtain further insights into how cells of different metastatic potential react to treatment, we evaluated the effect of an ADT agent like Enzalutamide on the cell lines of varied metastatic states, focusing on PSA secretion as a measure of therapeutic response. The influence of androgen stimulation and inhibition was evaluated on the cellular expression of PSA and PSMA (Figure 1). The cells were treated with R1881, and Enzalutamide as described in Section 2.3.1. LNCaP cells displayed PSA and PSMA expression in the untreated state (control), as shown by the green and red staining, respectively (Figure 1B,C). With androgen stimulation, an increase in both expressions was observed, as evidenced by the enhanced cytoplasmic staining (Figure 1F,G), suggesting the androgen sensitivity of LNCaP cells. However, PSA and PSMA expressions decreased after inhibition (Figure 1J,K) indicating that PSA and PSMA expression in LnCAPs are responsive to Enzalutamide (ADT). A difference in cell number (Figure 1A,E,I) observed between the conditions can be attributed to the stimulation using R1881 (0.5 nM). Previous studies have indicated the bi-phasic effect of R1881 on cell proliferation where concentrations between 0.1 and 1.0 nM promote cell growth and increase the percentage of cells in the G1 phase compared to those in G2/M/S phase, while the opposite is observed at concentrations higher than 1 nM [8,9].

Apart from LNCaP, the other cell lines (PWR-1E, RWPE-1, RWPE-2, PC3, 22Rv1) showed little to no visible PSA and PSMA expression in the untreated, stimulated, and inhibited conditions (Supplementary Figures S4–S8).

### 3.2. PSA Secretion from Single-Cell LNCaP in Response to Androgen Treatment

After analyzing the cellular PSA and PSMA expressions on the cell lines, we evaluated PSA secretion to determine whether the secretion correlates to the cellular expression on the cell lines. To evaluate the PSA secretion from the PCa cell lines, the cells were seeded on the anti-PSA coated PVDF membranes for 24- and 48 h timepoints as described in Section 2.4.2. The PSA spots captured were visualized using the immunofluorescence staining and quantified with the ImageJ software as described in Section 2.4.3 and Section 2.4.4. The secretions from every cell line were normalized to the background and the secretion from HepG2 cells (Supplementary Figure S9, Supplementary Table S3).

**Figure 2:**
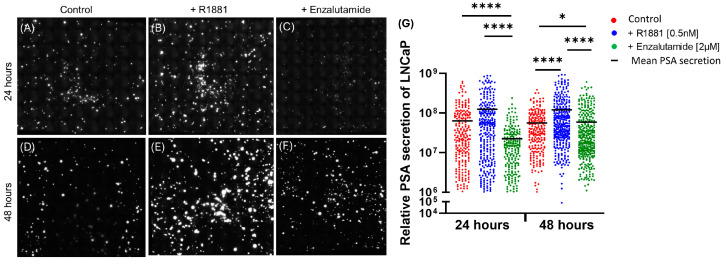
Effect of androgen treatment on PSA secretion from LNCaP cells. (**A**) 2000 LNCaP cells were seeded without any stimulus for 24 h. (**B**) 2000 cells LNCaP cells stimulated with R1881 [0.5 nM] were seeded, and secretion was captured 24 h after stimulation. (**C**) Overnight stimulated LNCaP cells with R1881 [0.5 nM] were treated with Enzalutamide [2 µM], and secretion was captured 24 h after inhibition. (**D**) 2000 LNCaP cells were seeded without any stimulus for 48 h. (**E**) 2000 cells LNCaP cells stimulated with R1881 [0.5 nM] were seeded, and secretion was captured 48 h after stimulation. (**F**) Overnight stimulated LNCaP cells with R1881 [0.5 nM] were treated with Enzalutamide [2 µM] and secretion was captured 48 h after inhibition. (**G**) Scatterplot showing relative PSA secretion (in arbitrary units) from single LNCaP cells after 24 and 48 h (N = 3). * *p* < 0.05, **** *p* < 0.0001, according to Kruskal–Wallis and Dunn’s post hoc test.

To illustrate the PSA spots, the PVDF membranes with the captured secretion from LNCaP are shown below as an example. At the 24 h and 48 h timepoints, PSA secretion was elevated following stimulation with R1881 (Figure 2B,E). The spots appear brighter and bigger than the untreated (control) sample (Figure 2A,D). In contrast, Enzalutamide inhibited PSA production as the spots captured on the membrane appeared dimmer and smaller (Figure 2C,F) than the untreated and stimulated cells. Secretion spots from the 48 h timepoint appeared brighter than the respective conditions in the 24 h timepoint.

The values for PSA in arbitrary units (A.U.) (mean intensity of spot * area of spot) secreted by individual LNCaPs after 24 and 48 hours are shown in a scatterplot in Figure 2G and Supplementary Table S4. The scatterplot of the secretions from the individual experiments is indicated in Supplementary Figure S10. The Kruskal–Wallis test was conducted to compare the secretion levels in the control, stimulated, and inhibited groups. The median (IQR) relative secretion of the control groups for 24 h was 2.31 × 10^7^ (7.34 × 10^6^–8.54 × 10^7^) and 3.33 * 10^7^ (1.33 × 10^7^–7.6 × 10^7^) for 48 h, indicating no significant change in secretion with increase in time.

Stimulation with R1881 led to a significant increase in the PSA secretion at 48 h (**** *p* < 0.0001) with median (IQR) relative secretion of 4.79 * 10^7^ (2.42 × 10^7^–1.24 × 10^8^), whereas the increase in PSA secretion at 24 h was not considered significant with median (IQR) values of 4.58 × 10^7^ (6.58 × 10^6^–1.09 × 10^8^). Inhibition with enzalutamide led to a significant decrease in secretion with median (IQR) values of 1.39 × 10^7^ (4.29 × 10^6^–2.87 × 10^7^) for 24 h (**** *p* < 0.0001), and 2.17 × 10^7^ (1.02 × 10^7^–6.19 × 10^7^) for 48 h (**** *p* < 0.0001). The decrease in secretion was greater at the 24 h timepoint compared to the 48 h timepoint, indicating the reduced effect of the drug after 24 h. This is supported by the significantly larger decrease in secretion at 24 h in the enzalutamide group compared to the control group (**** *p* < 0.0001), in contrast to the smaller difference observed at 48 h (* *p* < 0.05).

**Figure 3:**
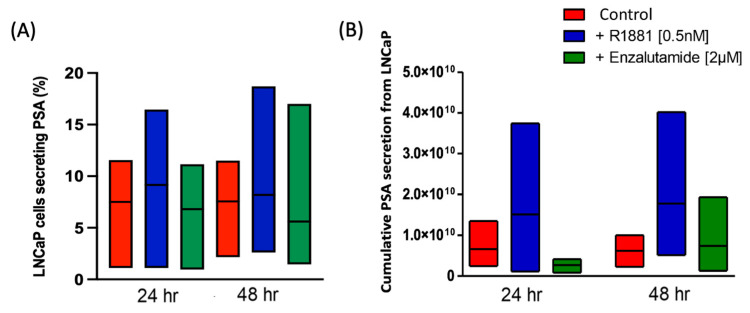
Effect of androgen treatment on PSA secreting LNCaPs. (**A**) Percentage of PSA-secreting LNCaP cells after 24 h and 48 h of androgen stimulation (blue) and inhibition (green) (N = 3). (**B**) Cumulative PSA secreted by LNCaP cells upon androgen activation and inhibition after 24 h and 48 h (N = 3).

The percentage of secreting cells also varied across the conditions (Supplementary Table S5). Of the 2000 cells seeded on the membrane, the percentage of secreting cells at the 24 h timepoint was a median (IQR) of 7.5% (1.11–11.55%) in the control, 9.15% (1.11–16.45%) in the R1881 stimulated, and 6.8% (0.95–11.15%) in the inhibited group. At 48 h, the percentage of secreting cells was 7.55% (2.15–11.50%) in the control, 8.2% (2.6–18.70%) in the R1881 stimulated, and 5.6% (1.45–17%) in the inhibited group. The percentage increase in secreting cells was almost minimal at 1.65% and 0.65% at 24 and 48 h, respectively. Similarly, androgen inhibition led to a decrease in the percentage of secreting cells of 2.35% and 2.6% at the 24 and 48 h timepoints, respectively (Figure 3A).

The total PSA secretion from 2000 LNCaP was determined by combining the PSA values of the individual cells (Figure 3B and Supplementary Table S6). The Kruskal–Wallis test was performed to compare the cumulative PSA secretion (in A.U.) across the control, stimulated, and inhibited groups. The control samples showed no significant differences between the 24 h and 48 h timepoints, with median (IQR) values of 3.99 × 10^9^ (2.45 × 10^9^–1.35 × 10^10^) at 24 h and 6.37 × 10^9^ (2.30 × 10^9^–1.01 × 10^10^) at 48 h, suggesting consistent secretion over time. PSA secretion increased with stimulation, though not significantly, with median (IQR) values of 6.83 × 10^9^ (1.14 × 10^9^–3.75 × 10^10^) at 24 h and 7.90 × 10^9^ (5.19 × 10^9^–4.02 × 10^10^) at 48 h. Androgen inhibition demonstrated a larger decrease in PSA secretion at 24 h with median (IQR) values of 3.22 × 10^9^ (8.47 × 10^8^–4.15 × 10^9^) compared to 1.64 × 10^9^ (1.33 × 10^9^–1.94 × 10^10^) at 48 h, further suggesting that the effect of Enzalutamide diminishes over time.

Overall, the extent of changes in secretion varied between experiments, reflecting the heterogeneous nature of secretion from LNCaP cells. There were no distinct differences in the PSA secretion between the 24 and 48 h timepoints, suggesting that the secretions stabilized over time. Altogether, these findings indicate that PSA secretion by LNCaPs is modulated by both androgen stimulation and ADT, with distinct effects observed at the single-cell level for each treatment.

The PSA spots detected from other cell lines were minimal and were consistent with the limited PSA cellular expression observed in Supplementary Figures S4–S8. Due to few secretion spots (possibly due to the detection limit), the effect of stimulation and inhibition could not be measured effectively. The scatter plot showing the PSA secretion for 24 and 48 h and the percentages of secreting cells in response to stimulation and inhibition from RWPE-1, RWPE-2, PWR-1E, 22Rv1 and PC3 are shown in Supplementary Figures S11 and S12 and Supplementary Table S7.

To confirm which of the cell lines secrete PSA, an ELISA test was performed with and without stimulation to quantify secretion from the cell lines of RWPE-1, RWPE-2, PWR-1E, 22Rv1 and PC3 as shown in Supplementary Figure S13 and Supplementary Table S8. The PSA secretion levels for LNCaPs were found to be a median (IQR) of 33.94 pg/cell/day (32.30–35.57) and increased by a 2.6-fold to 89.43 pg/cell/day (87.94–90.91) after stimulation with R1881. While a clear trend was observed with stimulation, the increase in LNCaP cells was not significant. The PSA secretion from 22Rv1 cells was found to be a median (IQR) of 0.38 pg/cell/day (0.37–0.38). After stimulation with R1881, PSA secretion increased negligibly to 0.42 pg/cell/day (0.41–0.44) suggesting androgen insensitivity. The other cell lines did not exhibit detectable PSA secretions under any of the treatment conditions.

**Figure 4:**
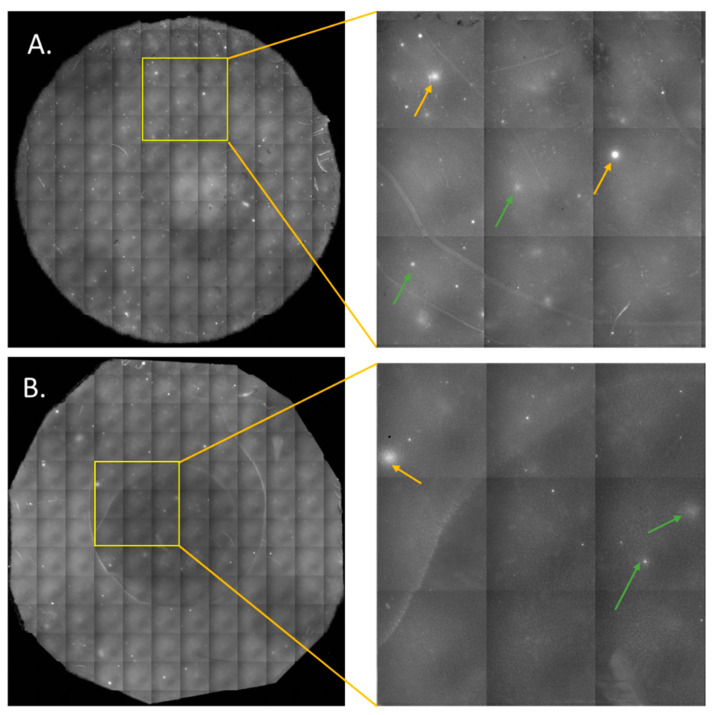
PSA secretion from circulating tumor cells (CTC) from mCNPC patients (N = 2). Panels (**A**,**B**) show examples of membranes containing PSA secretion spots from the two patients on the left and a magnified image of the yellow region of interest on the right. PSA secretion is captured from the CTC of the DLA samples (1 × 10^8^ cells) incubated on the membrane for 24 h. Heterogeneity is observed in secretions due to the presence of bright spots (indicated in yellow) and dim spots (indicated in green) in both patient samples. Membranes imaged with FITC at 100 ms exposure.

To further validate the PSA secretion observed from the LNCaP cells, circulating tumor cells (CTCs) were enriched from DLA aliquots of 2 × 10^8^ WBCs from two mCNPC patients using the CellSearch system as per the methods described in Section 2.7. The enriched CTCs, equivalent to 1 × 10^8^ WBCs, were seeded on the membranes to capture PSA secreted over 24 h. The images of the membranes from the two patients shown in Figure 4 reveal the presence of PSA spots in the CTCs derived from both patient samples. Secretion was found to be heterogeneous due to the presence of bright and dim spots indicated by yellow and green arrows, respectively, thereby validating the assay’s effectiveness in measuring PSA secretion at the single-cell level and further confirming the PSA heterogeneity observed using LNCaP cells.

### 3.3. Effect of Cell Cycle on the Production of PSA

**Figure 5:**
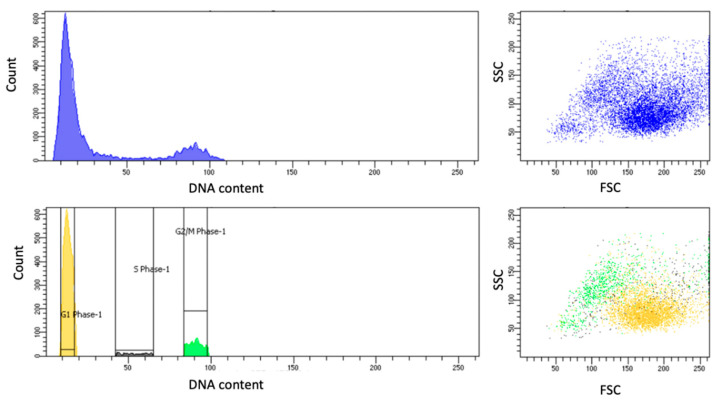
FACS plots for analyzing cell cycle phases and cellular granularity in LNCaP cells. The left panels display histograms of DNA content with the initial plot showing an unimodal distribution indicative of a homogeneous population, and the subsequent plot segmented into G1, S, and G2/M phases, highlighted in yellow, green, and gray, respectively. The right panels are scatter plots correlating forward scatter (FSC), which indicates cell size, with side scatter (SSC), which reflects internal complexity or granularity. The top scatter plot shows the entire cell population, while the bottom plot highlights cells gated from specific cell cycle phases, color-coded to match the histogram.

To investigate the heterogeneity in the PSA secretion from LNCaP cells (indicated in Figure 2), the cell cycle of the LNCaPs was examined to understand how the secretion varies during different cell cycle phases. The LNCaP cells were stained with Hoechst 3342 and analyzed via the FACS in the DAPI channel as per the protocol mentioned in Section 2.5. The analysis with FACS yielded a plot (Figure 5) representing the DNA content of the cells. The single cells, represented in blue, accounted for 85% of the total cells analyzed. By establishing gates on the DNA content histograms, cells in the G1, S, and G2/M phases, indicated by distinct peaks, were sorted into tubes for further cellular analysis following cytospin and secretion studies. Among the single-cell population, the highest proportion of cells was in the G1 phase (46%) followed by 9% in the G2/M phase and 4% in the S phase. The remaining 26% of cells were in transition between phases and did not fall precisely within the G1, S, and G2/M gates, and therefore, they were not sorted for further analysis.

#### 3.3.1. Morphology of Cells in the G1, S, and G2/M Phase

To visualize the morphology of the cells in each cell cycle phase, the cells were sorted as shown in Figure 5 and deposited on glass slides by cytospin. Once deposited onto the slides, the cells were fixed using a mounting medium containing DAPI to stain the nuclei, allowing for visualization of the DNA content of the cells.

**Figure 6:**
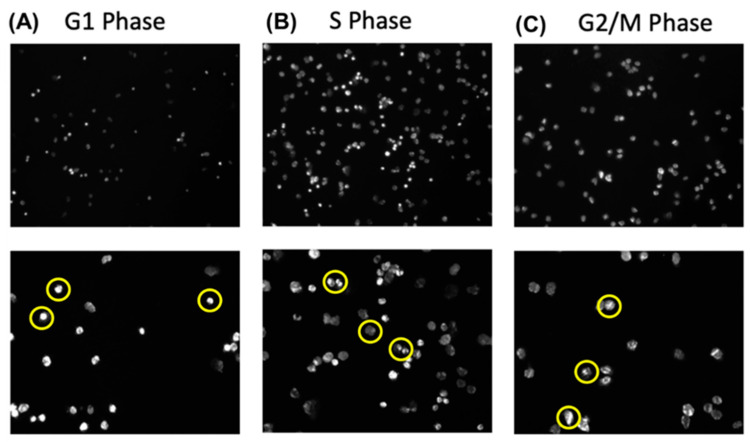
Hoechst stained LNCaP cells in G1, S and G2/M phases. LNCaP are FAC sorted into the different cell cycle phases, followed by cytospin and visualization in the DAPI channel at 10× (first row) and 20× magnification (second row). Examples of cells (in yellow) indicate a single homogenous staining in (**A**) G1 phase. (**B**) The S phase shows irregular staining and beginning DNA replication. (**C**) The G2/M phase shows the presence of a mitotic spindle indicating cell division.

As shown in Figure 6, the cells sorted for the G1 phase appear predominantly as single cells with a single nucleus, consistent with cells in the initial growth phase of the cell cycle, as highlighted by the yellow circles.

The DAPI staining of the cells sorted for the S phase exhibits some irregular staining as the cells are in the beginning stages of DNA replication and cellular division. The cells sorted for the G2/M phase display multiple nuclei or a single nucleus with doubled DNA content, indicating the final stages of the cell cycle before mitosis [10,11].

#### 3.3.2. PSA Secretion in Different Cell Cycle Phases

**Figure 7:**
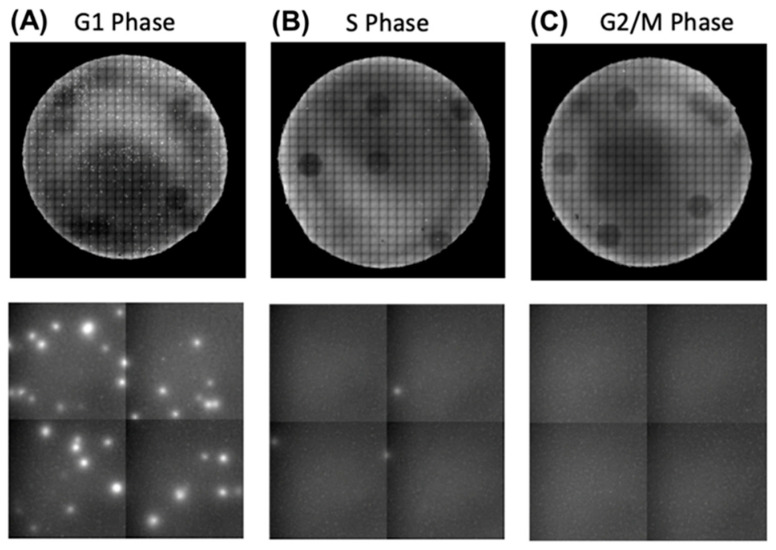
PSA secretion from LNCaP cells sorted into G1, S, and G2/M phase on PVDF membranes. (**A**) Highest PSA spots observed from cells in the G1 phase, (**B**) few secretion spots seen in S phase and (**C**) no secretion was observed in G2/M phase. The second row indicates magnified images of an area on the membranes presented in the first row.

After sorting and verifying that the cells have been sorted into their respective phases, the secretion patterns of PSA for each cell cycle phase (G1, S, and G2/M) was evaluated. The images analyzed using ImageJ provided distinct secretion profiles across the different phases, as illustrated in Figure 7.

From the membrane images, it was evident that cells in the G1 phase were actively secreting PSA, as indicated by the distinct spots. In contrast, cells sorted into the S and G2/M phases did not exhibit significant PSA secretion, with minimal or no spots detected on the membranes. The findings suggest that PSA secretion is predominantly active during the G1 phase, while cells in the S and G2/M phases contribute negligibly.

### 3.4. Identification of Potential Biomarkers Using Proteome Array

While PSA remains a key biomarker in PCa, its secretion and treatment response could be studied only in the LNCaP cell line, which limits our understanding of the other metastatic states where tumor heterogeneity is prominent. To represent both the castration-naïve and castration-resistant metastatic states, cell lines LNCaP and 22Rv1 were chosen. Since both these cell lines secrete PSA as indicated by the ELISA results (Supplementary Figure S12), a PSA non-secreting metastatic cell line of PC3 was also included to explore differences in secretome production between the CNPC and CRPC states. The culture medium derived from LNCaP, PC3, and 22Rv1 cells was tested for other potential biomarkers using the Proteome Array per the manufacturer’s instructions. A control sample consisting of the culture medium without serum was analyzed concurrently. The antibody spot design containing the array coordinates and the proteins corresponding to these coordinates are mentioned in the Appendix A.

**Figure 8:**
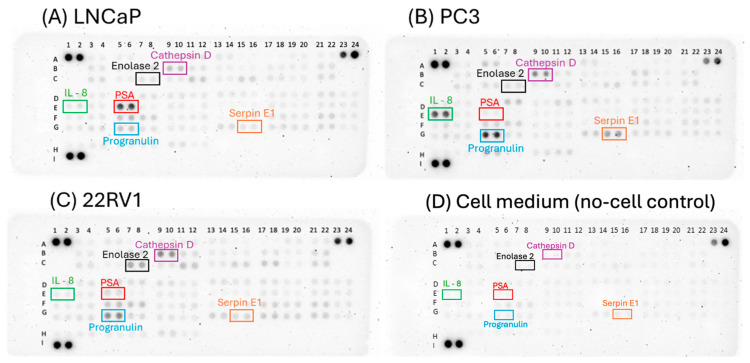
The proteome array data from PCa cell lines (**A**) LNCaPs (**B**) PC3 (**C**) 22Rv1 and (**D**) cell medium (no-cell control). Each array highlights the spots (in duplicates) corresponding to the proteins detected in the supernatant. The most visible spots are identified in colored boxes and represent the proteins IL-8 (green), PSA (red), Enolase 2 (black), Cathepsin D (purple), Serpin E1 (orange), and Progranulin (blue).

**Figure A2:**
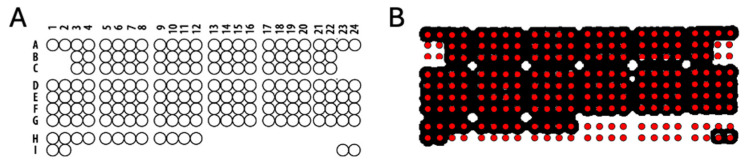
Schematic structure conversion to digital array overlay. (**A**) This schematic provides an overview of the structure of the protein array. It serves as a reference for computing the reference matrix, a crucial step in the analysis process. (**B**) This is the computed reference matrix. The red dots represent all points ranging from the coordinates [A..I] and [1..24]. The algorithm includes all points within the matrix for simplicity. However, it is important to note that in the final analysis, only the red dots within the black area are pertinent and contribute to the intended analysis.

The spots on the array were visualized for LNCaP, PC3, 22Rv1, and the culture medium as indicated in Figure 8. To analyze the proteome array data, the pixel intensities of the spots were calculated to compare the protein secretions between the different cell lines. The pixel intensities along with the mean ± standard error of the mean (SEM) obtained from the cell lines for all proteins are provided in Supplementary Table S9. The quantification protocol, antibody spot design containing the array coordinates (Appendix Figure A2), and the proteins corresponding to these coordinates are mentioned in the Appendix A. The levels of protein secretion (measured as pixel intensities) were compared across the LNCaP, PC3 and 22Rv1 cell lines, and the median (IQR) values were reported.

**Figure 9:**
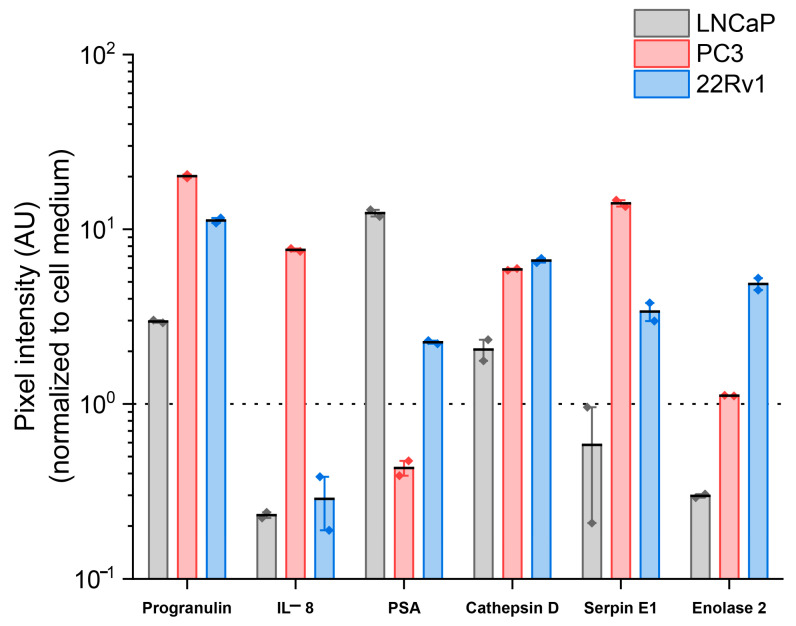
Quantification of secreted proteins from cell lines LNCaP, PC3, 22Rv1. The y-axis represents pixel intensities of the spots (arbitrary units) normalized to the cell medium (represented as a black dotted line). The intensities extracted from the Progranulin, IL-8, PSA, Cathepsin D, Serpin E1, and Enolase 2 are represented as mean ± standard error of the mean (SEM). The black dashed line on the bars represents the median.

In Figure 9, the progranulin secretion (in A.U.) was observed to be higher in castration-resistant cells lines 22Rv1 and PC3 with a median (IQR) of 11.23 (10.85–11.61) and 20.12 (19.66–20.59), respectively, compared to castration-sensitive cell line LNCaP with a value 2.96 (2.91–3.02). PC3 showed the highest concentration of IL-8 with 7.61 (7.48–7.75) and was negligible in 22Rv1 and LNCaP with 0.29 (0.19–0.38) and 0.23 (0.22–0.24), respectively. PSA is predominantly expressed in LNCaPs with 12.38 (11.82–12.93) followed by the 22Rv1 cells with 2.26 (2.21–2.30), a trend previously seen in ELISA. Cathepsin D expression is more predominantly secreted by 22Rv1 with 6.62 (6.44–6.80) and PC3 with 5.89 (5.82–5.96) compared to the LNCaP with 2.05 (1.77–2.33). Serpin E1 a resistance-associated gene [12], showed higher secretions in PC3 with 14.08 (13.48–14.67) and 22Rv1 with 3.38 (2.98–3.78) compared to the LNCaPs with 0.58 (0.21–0.96). Enolase 2 or neuron-specific enolase (NSE) is secreted only from 22Rv1 with 4.86 (4.48–5.24) and was negligible in PC3 with 1.12 (1.11–1.12) and LNCaP with 0.3 (0.29–0.3). Of the identified proteins, Progranulin and Cathepsin D showed high secretions in castration-sensitive and castration-resistant cell lines, highlighting their potential as biomarkers that should be further investigated in a metastatic cancer setting. Although the overall results showed clear trends in marker expression, the statistical analysis did not reveal significant differences between the cell lines expressing the proteins.

### 3.5. Validation of Biomarkers Using Dot Blot

Finally, we performed the dot blot to visualize the secretion of cathepsin D and progranulin by LnCAP, PC3, 22Rv1, and RWPE-1 using an undiluted and diluted (1:10) sample of the conditioned medium dotted on the membrane. The dot blot assay showed high progranulin and cathepsin D expression in all metastatic PCa lines and much lower expression in benign RWPE-1 cells (Supplementary Figures S14 and S15), which aligns with the proteome array.

## 4. Discussion

Despite the advances in PCa therapy, most patients with metastatic disease present therapy resistance and tumor heterogeneity. Understanding the disease on a single-cell level using secretome biomarkers can help to stratify patients and optimize therapy. In this study, we aimed to examine different PCa cell lines to assess the phenotypic response of PCa cells in response to R1881 and enzalutamide treatment using PSA as a biomarker and assess the differences in the metastatic disease states. Moreover, we assessed the influence of the cell cycle phase on PSA secretion and attempted to identify new biomarkers besides PSA. Lastly, to demonstrate that prostate cell lines can be used to study the secretome of prostate cancer cells, we showed that similar PSA secretion patterns can be observed in CTC obtained from two mCNPC patients.

To understand the effect of androgen stimulation and ARSIs in the different disease stages, the cell lines of LNCaP and RWPE-2 (CNPC), RWPE-1 and PWR-1E (benign), 22Rv1, and PC3 (CRPC) were phenotypically characterized for the presence and secretion of PSA. Stimulation of the androgen receptor showed increased presence and secretion of PSA from the LNCaP, and the effect Enzalutamide could be observed with the decrease in PSA expression and secretion, thereby supporting the properties of a hormone or castration-sensitive cell line [4,13]. While it is known that AR stimulation decreases PSMA expression, the opposite was observed in the LNCaP cells. This anomaly will need to be validated by looking at the PSMA mRNA of the stimulated cells. The RWPE-2 cell line, however, does not share this characteristic with LNCaPs, as the secretion (bulk or single cell) shows no change upon stimulation or inhibition. While no studies have measured the secretion from RWPE-2, the percentages of PSA-secreting cells and the relative secretion of PSA graphs (Supplementary Figures S11 and S12) of the RWPE-2 are comparable to the negative control cell line HepG2, making it clear that secretion from this cell line is AR independent.

The benign cell lines of PWR-1E and RWPE-1 are known to be androgen-responsive and express low amounts of PSA but cannot be easily visualized as seen in Supplementary Figures S4 and S5 [14,15]. Studies from Bello D. et al. and Webber M. et al. show that with stimulation (for 6 days with 5 nM R1881), AR activity and PSA expression is induced [14,16]. Our results show dim expression of PSA 48 h after stimulation and 24 h after AR inhibition with Enzalutamide. This may indicate that Enzalutamide does not affect the benign cell lines. However, the low expression of cellular PSA can be attributed to shorter stimulation time and lower concentration of the stimulant. The cell lines of 22Rv1 showed low levels of PSA expression (Supplementary Figure S6), which is contradictory to findings in the literature [15,17]. Low levels of PSMA expression can be attributed to the fact that only 20–30% of the cells express PSMA and are heterogeneous in its expression [18,19]. Results from ELISA indicate the presence of secreted PSA in the supernatant however, the amount and the intensity of spots detected from single cells on the membrane are comparable to HepG2, indicating no secretions. While the effect of R1881 is seen on the bulk PSA secreted (Supplementary Figure S13), the same is not visible from single cells (Supplementary Figure S11) which can be due to reduced viability of cells on the membrane or differences in secretion intensity that the membranes are not sensitive enough to capture. PC3 cells did not express nor secrete PSA, were independent to AR stimulation or inhibition as shown previously [20,21].

The cells of both LNCaP and 22Rv1 can be used as models to study the differences in CNPC and CRPC using PSA as a biomarker, However, LNCaP proves to be a better candidate and is the only cell line where we observed PSA secretions and heterogeneity in its secretion at the single-cell level. Many studies explore tumor heterogeneity through cellular PSA expression [22], however, few have investigated how secretion patterns vary in cell phases and how cell phases influence PSA heterogeneity. Using FACS, we successfully identified distinct peaks corresponding to each cell cycle phase, enabling accurate sorting of LNCaP cells into the G1, S, and G2/M phases. The analysis of PSA secretion revealed that only cells in the G1 phase secreted significant levels of PSA while, cells in the S and G2/M phases did not secrete detectable amounts of the protein (Figure 7). Enzalutamide targets the AR signaling pathway and induces cellular senescence by arresting the cells in the G1 phase of the cell cycle [23,24]. This reduces the PSA production as observed in the castration-sensitive cells of LNCaP (Figure 2). The secretion seen post Enzalutamide treatment can account for cells that have not achieved cell cycle arrest or are transitioning from G1 to S phase. Long-term androgen deprivation can lead to the metastatic stage of CRPC driven by EMT, thereby causing AR re-activation and PSA production. Using the G1 phase of the cell cycle, ARSIs can be tested to observe the secretion patterns of the biomarker of PSA in the CRPC state as well. Using the cell cycle synchronizing agents such as Ciclopirox olamine, Aphidicolin, and Palbociclib, the cells of 22Rv1 representing the CRPC stage can be synchronized into G1 phase to improve PSA secretion. Achieving synchronization in LNCaP and 22Rv1 can be useful for comparing proteome secretion across cell cycle phases and observing the response to therapeutic agents. A higher PSA secretion can be observed from R1881 stimulated cells as shown in Figure 2 and 3B. R1881 used at low concentrations (0.1–1 nM) can help maintain the cells in the G1 phase [8,9] and enable testing of ARSIs to measure PSA response.

Relying solely on PSA as a biomarker can limit the use of cell line-based models as we observed little to no secretions from all cells except LNCaP cells. To overcome this, we identified multiple protein markers using the Proteome array. Progranulin could be visualized at a higher intensity from the metastatic cell lines than the benign cell line of RWPE-1 (Supplementary Figure S14). A growth factor commonly elevated in cancer, progranulin is known to present in the liquid biopsy (serum) of younger patients (<65 years) with PCa and can show potential as a prognostic biomarker since their elevated levels were linked to shorter overall survival in patients [25,26]. Likewise, Cathepsin D demonstrated higher protein secretion from the metastatic cell lines compared to RWPE-1 (Supplementary Figure S15). Although Cathepsin D is not a widely explored marker, it is recognized as a good predictor of survival [27,28] and warrants further investigation due to its presence in the secretome of PCa cells. Additionally, three more proteins should be investigated further in their capacity as CRPC markers namely, IL-8, Serpin E1, and Enolase. IL-8, an inflammatory cytokine is known to be a contributor in the CRPC stage and linked to shorter overall survival in patients [29,30]. Serpin E1 is recognized for its role in promoting tumor progression and metastasis and its expression has been associated more with cells resistant to docetaxel compared to those that are sensitive [12]. This also correlates with secretion data as higher secretions were observed from PC3 and 22RV1 compared to the negligible levels in LNCaP. Enolase2 is a marker for neuroendocrine differentiation (NED). 22Rv1 exhibiting Enolase can make a useful model to understand the role of NED that subsequently leads to the development of resistance to prolonged therapy [31]. Although Cathepsin D and Progranulin show prominent secretion in metastatic cell lines, they fail to show differences between the cell lines of different metastatic stages. This highlights the need for a combination of biomarkers to address the limitations of using these secretome markers as standalone indicators.

A key limitation in this study is that the membranes used to observe secretion are effective only for high PSA-producing cell lines like LNCaP, and fail to distinguish between low PSA-producing cell lines and artifacts. Considering the presence of secretome markers from 22Rv1 and PC3, it is essential to investigate their secretion on a single-cell level to enhance the understanding of the aggressiveness of this disease state. Using a single-cell platform such as the VyCAP chip can address this limitation by improving the cell-membrane contact and protein capture [4,32], thereby enhancing the overall functionality of the assay. Moreover, including primary cell lines could broaden the scope of the assay and help in identifying other models beyond LNCaP that more accurately mimic the different stages of the disease. Another limitation is that this assay was validated using only two patient samples, which is insufficient to offer a comprehensive understanding of the secretion profiles of the tumor cells. A more robust analysis would require a larger sample size, including patients of different metastatic stages, to investigate secretion patterns and explore how patient-derived CTCs differ from cell line models. While using CTCs can improve the validity of the assay to measure secretion patterns, it also presents certain challenges. The low frequency of CTCs makes it difficult to isolate cells into distinct phases, based on their cell cycle. As a result, testing ARSIs in the S1 phase of the CTCs may prove challenging, necessitating reliance on the cell line models for such studies. Lastly, the identification of the biomarkers using the proteome array was based on only three cell lines, which limits the applicability of these biomarkers in other models. To validate their relevance, the proteome array can be tested using patient samples to confirm whether the identified biomarkers are also secreted in clinically relevant models. Further validation can be carried out by investigating protein secretion at the single-cell level in response to therapy. This can indicate whether these biomarkers, alongside PSA, show potential for clinical use.

Future work can explore multiplexing to capture and visualize multiple secreted proteins. For ELISpot, multiplexing capabilities have been explored in the context of cytokine detection, where multiple cytokines can be detected simultaneously in a single assay [33]. However, multiplexing for secretome markers in PCa, particularly for proteins such as PSA and other potential biomarkers, has not been extensively reported. Similarly, while FACS allows for the analysis of multiple biomarkers simultaneously on a single-cell level, its application for secretome markers in PCa is not well documented. Therefore, the absence of established multiplexing methods for secretome markers in PCa underscores the need for further research in this field. Developing multiplexing techniques on a single-cell platform could significantly enhance our ability to understand the complex secretome profiles of PCa cells [34], potentially leading to improved diagnostic and therapeutic strategies.

## 5. Conclusions

We demonstrated notable variability in PSA secretion from individual LNCaP cells in response to anti-androgen treatments, and a cell cycle-dependent pattern in secretion. These findings emphasize the importance of considering cell cycle phases to obtain accurate data on proteome secretion patterns which can aid in optimizing therapies. Cathepsin D and Progranulin were identified as potential secretome markers due to their significant presence in CNPC and CRPC states. Further investigation of these potential secretome markers alongside PSA using single-cell and multiplexing platforms can help to improve our understanding of tumor complexity and develop strategies to combat therapy resistance.

## Figures and Tables

**Figure A1:**
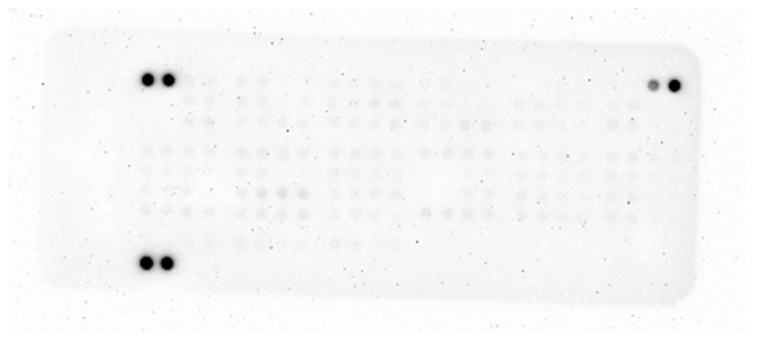
Picture of a single to-be-analyzed protein array membrane.

**Figure A3:**
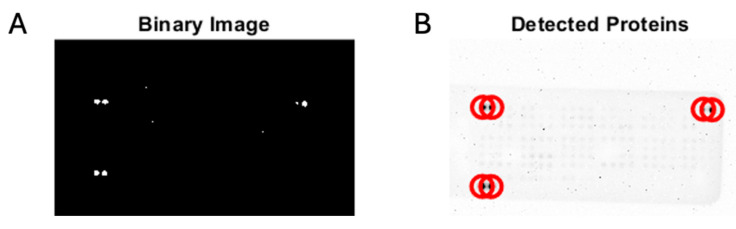
Image processing of proteome array (**A**): Image processing result for detecting the reference spots within the to- be-analyzed protein array membrane (**B**): Found possible reference spots marked with a red circle.

**Figure A4:**
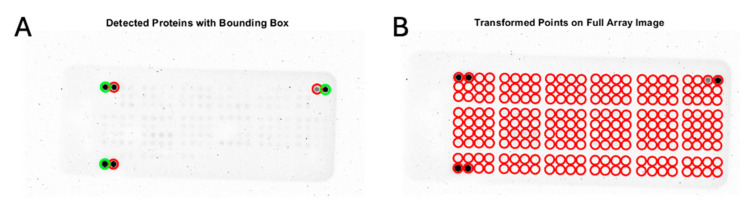
Schematic structure conversion to digital array overlay. (**A**) Detected reference spots marked with a green circle. (**B**) The to-be-analyzed protein array membrane with the reference matrix fitted over.

**Figure A5:**
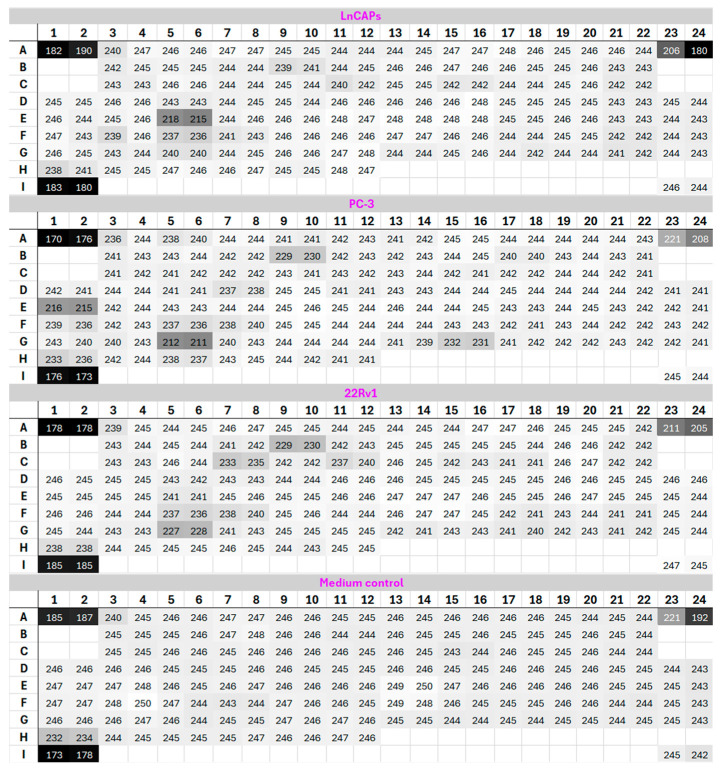
Intensity matrix resulted from the Protein Analyzer Software. Here, the intensity values are converted into color codes to improve the visualization of the values mapped from 0 to 255.

**Figure A6:**
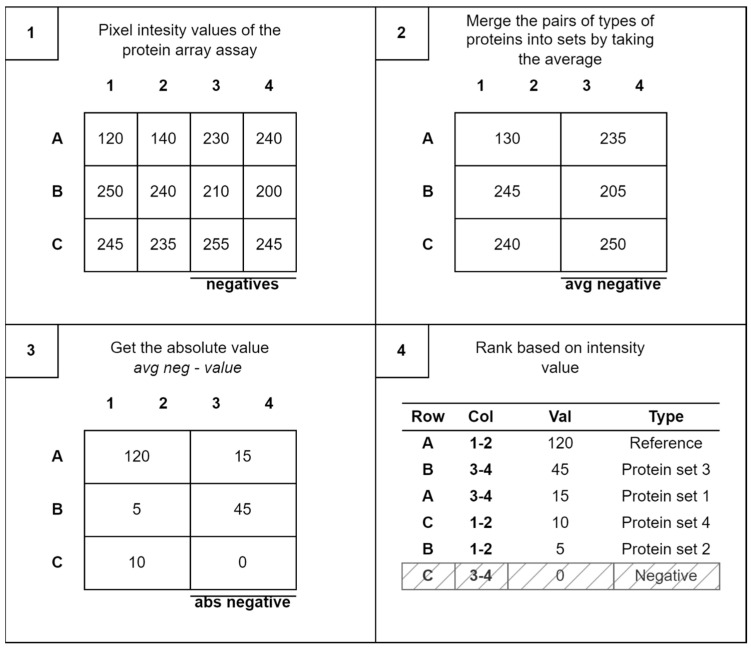
Flowchart illustrating the analysis of proteome array results. The data were first collected as pixel intensity values, with example values shown in each grid. C3,4 being an example for the negative reference, which is used to obtain the absolute values of the results and ensure accuracy when comparing various sets of results.

## Data Availability

This article includes all data generated in this study. The data are found in the form of figures and Supplementary Information in this paper. The lead contact can provide information for all relevant data and resources upon request.

## Supplementary Materials

The following supporting information can be downloaded at: https://www.mdpi.com/article/10.3390/cancers17050721/s1, Table S1: Characteristics of PCa cell lines; Table S2: Antibodies used for immunofluorescence staining, single-cell secretion staining, ELISA and dot blot assay; Table S3: HepG2 secretion and no cell secretion (A.U.) values from 24 h timepoint. Table S4: PSA secretion (A.U) values from LNCaP cells for 24- and 48 h timepoints; Table S5: Percentages of LNCaP cells secreting for 24- and 48 h timepoints; Table S6: Total PSA secretion from LNCaP for 24- and 48 h timepoints; Table S7: Relative mean PSA secretion values and percentage of secreting cells from PWR-1E, RWPE-1, RWPE-2, 22Rv1 and PC3 cell lines; Table S8: PSA secretions (picogram/cell/day) from cell lines; Table S9: Proteome Array pixel intensities (A.U) of the proteins secreted by the cell lines. Figure S1: Illustration showing the workflow to study the effect of stimulation and inhibition on cell lines PWR-1E, RWPE-1, RWPE-2, LNCaP, 22Rv1, PC3 and HepG2; Figure S2: Workflow of cell seeding and capturing of PSA on the PVDF membranes; Figure S3: Workflow of cell sorting into different cell cycle phases; Figure S4: Immunofluorescence staining in PWR-E1 cells; Figure S5: Immunofluorescence staining of RWPE-1 cells; Figure S6: Immunofluorescence staining of 22Rv1 cells; Figure S7: Immunofluorescence staining of PC3 cells; Figure S8: Immunofluorescence staining of RWPE-2 cells; Figure S9: Relative intensities from membranes seeded with HepG2 cells (after 24 h) and empty membranes; Figure S10: Scatter plot showing the effect of androgen treatment on PSA secretion from LNCaP cells; Figure S11: Single-cell PSA secretion from PWR-1E, RWPE-1, RWPE-2, 22Rv1 and PC3 (N = 3); Figure S12: Percentages secreting cells of PWR-1E, RWPE-1, RWPE-2, 22Rv1, PC3 and HepG2 after (A) 24- and (B) 48 h timepoints; Figure S13: Effect of androgen treatment on bulk PSA from cell lines PWR-1E, RWPE-1, RWPE-2, LNCaP, 22Rv1 and PC3; Figure S14: Dot blot to measure Progranulin from cell lines; Figure S15: Dot blot to measure Cathepsin D from cell lines.

## Appendix A

### Protein Analyzer Software

This appendix describes the workings of the Protein Analyzer Software. The tool was developed to analyze protein secretions by examining pixel intensity of each spot, which corresponds to a specific biomarker. The following sections explain the workings of the written code. The code is designed around an image of four membranes used in the protein array assay. For clarity, the focus will be on the process of analyzing a single array assay, as depicted in Figure A1.

Initially, a reference image of the assay’s array, as shown in Figure A2A, is loaded to establish the relative pixel positions of the proteins within the array. This facilitates the creation of a reference matrix containing the pixel coordinates of each protein within the array, depicted in Figure A2B. This matrix will be fitted over the to-be-analyzed protein arrays. From this matrix, we determine the relative positions of the upper-right, upper- left, and lower-left points. These will be later used for the fitting process.

Next, the image of the protein array membrane undergoes analysis. To align the reference matrix with the array’s membrane image, the upper-right, upper-left, and lower- left points must be identified. This involves utilizing image processing techniques to isolate the spots, as shown in Figure A3, focusing on the reference spots.

After detecting these spots, sorting algorithms are applied to determine the reference positions, such as the upper right. These are marked with a green circle in Figure A4A. Following this, a bounding box is applied to the reference points to facilitate their exclusion when identifying reference points on multiple protein array membranes.

With the reference points established for both the reference matrix and the to-be- analyzed protein array membrane, the geometrical difference between the two can be determined using the fitgeotrans function from MATLAB (v. R2024a) and stored in a transformation matrix. This transformation matrix is then applied to the reference matrix to fit it over the protein array membrane, as shown in Figure A4B, enabling the identification of pixel coordinates for each spot in the assay’s array membrane. Finally, with the pixel coordinates of all spots within the assay’s array determined, the intensity of each protein is computed. This involves calculating the average pixel intensity within a 5-pixel radius around each point, resulting in a matrix corresponding to the assay’s array (see Figure A5).

After acquiring the pixel intensities, the following steps are performed to process and analyze these data, as illustrated in the accompanying flowchart (Figure A6):

1. **Data Collection:** Pixel intensity values are extracted from the array images, representing the relative abundance of each protein.
2. **Normalization:** Values are normalized against a negative control to correct for background noise and variability in staining intensity.
3. **Threshold Setting:** A threshold is set to distinguish between meaningful signals and background noise, enhancing the reliability of the data interpretation.
4. **Data Compilation:** The processed data are compiled into a structured format for further statistical analysis and visualization.

The proteins corresponding to the coordinates (as shown in Figure 2A) can be referenced in the datasheet provided by the manufacturer of the Proteome Profiler Human XL Oncology Array.
