# Supplementary material for: Secretome Analysis of Prostate Cancer Cell Lines Reveals Cell Cycle-Dependent PSA Secretion and Potential Biomarkers
Source: Cancers (Basel). 2025 Feb 20;17(5):721. doi: 10.3390/cancers17050721 (PMC11899065; doi:10.3390/cancers17050721)
Supplement: Supplementary file 1 [file cancers-17-00721-s001.zip › Supplementary_Figures.pdf]

Supplementary Figures:

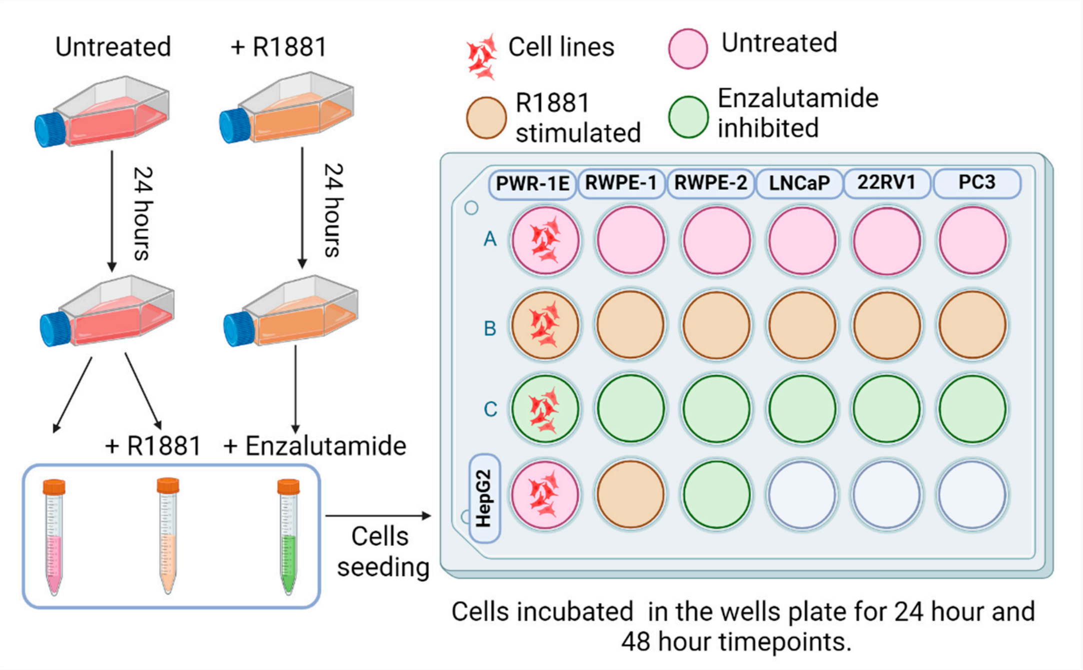

Figure S1: Illustration showing the workflow to study the effect of stimulation and inhibition on cell lines PWR-1E, RWPE-1, RWPE-2, LNCaP, 22Rv1, PC3 and HepG2. Two flasks of each cell line were prepared, of which one is treated with R1881 and the other remains untreated. After 24 hours, the cells of the untreated flask were split into two cell suspensions. One part remained untreated (pink tube); the other part was stimulated with R1881 (orange tube). Enzalutamide was added to the cells collected (in green tube) from the second flask which was treated overnight with R1881. Cells from the three tubes were seeded in the 24-well plate (as indicated by an example in the first column). Created by biorender.com

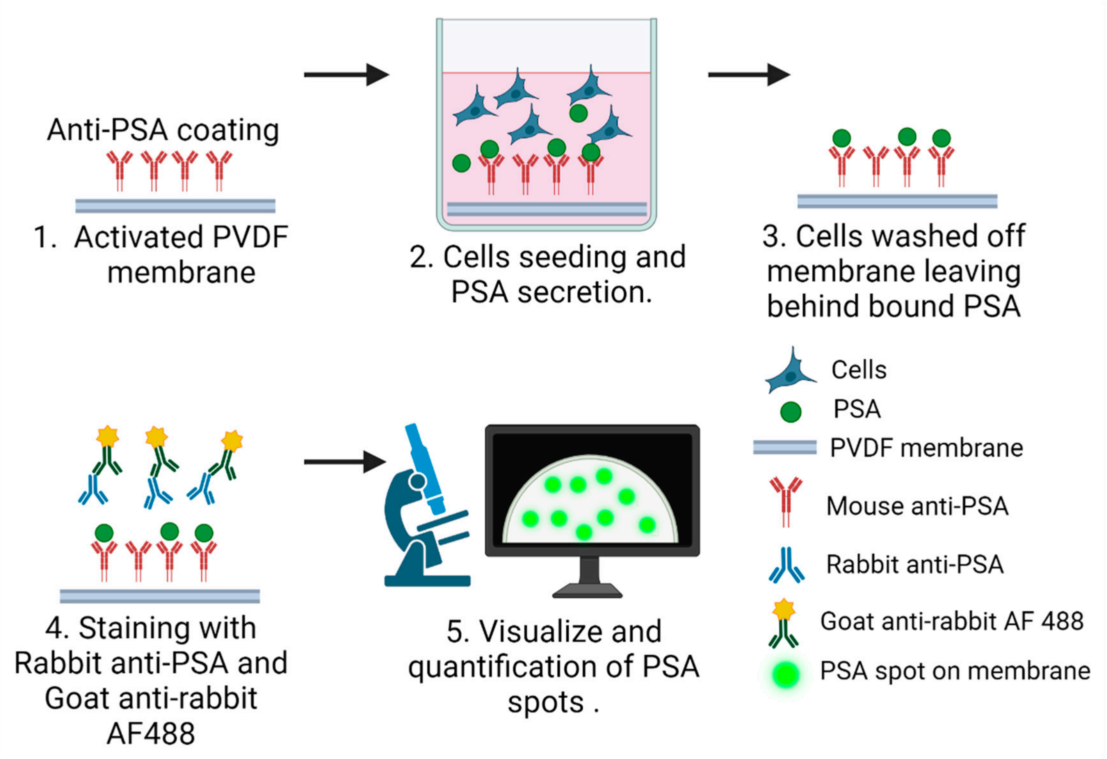

Figure S2: Workflow of cell seeding and capturing of PSA on the PVDF membranes. 1. The membranes were coated with capture antibody (anti-PSA) overnight. 2. Cell suspension of 2000 cells was seeded directly on the coated membrane in a wells plate and incubated overnight to collect secretion. 3. The cells are washed off leaving behind the secreted and captured PSA. 4. PSA spots stained with primary antibody rabbit anti-PSA, then with fluorescently labeled secondary antibody goat anti-rabbit AF488. 5. After staining, membranes were dried and scanned (FITC channel) using the inverted microscope followed by ImageJ software analysis. Created with Biorender.com.

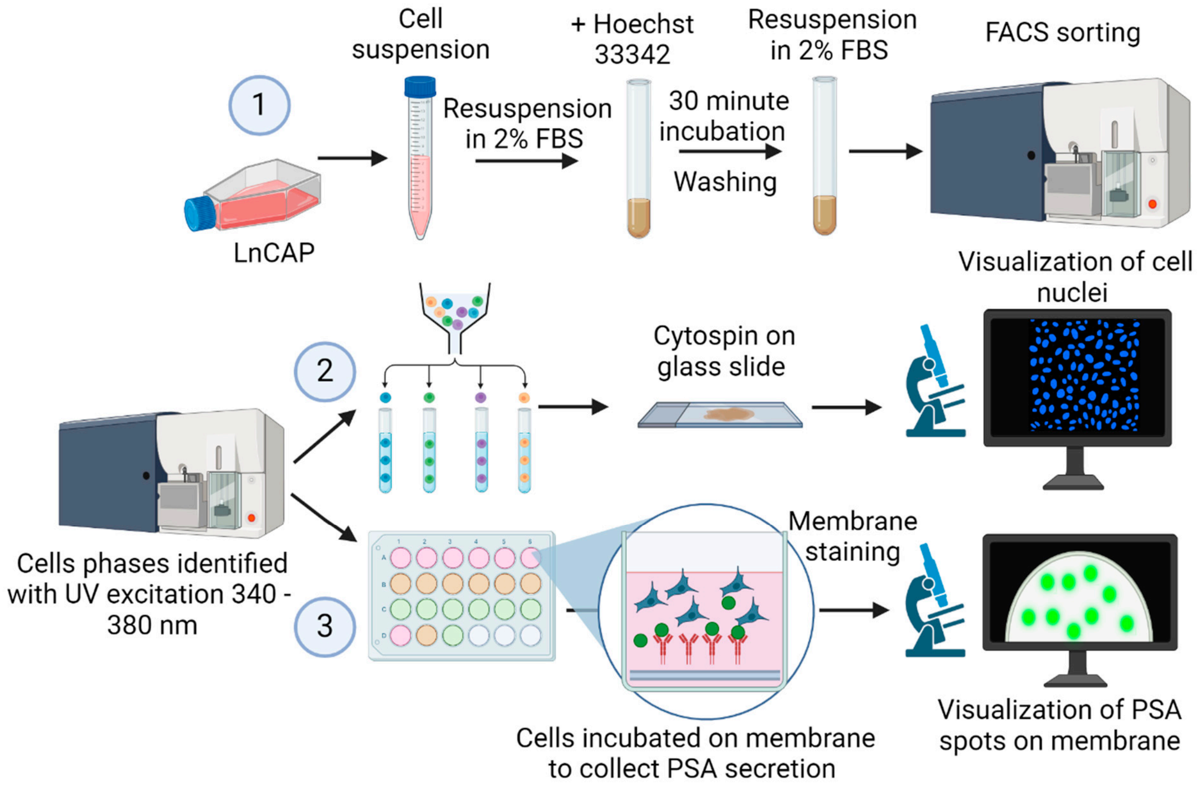

Figure S3: Workflow of cell sorting into different cell cycle phases. 1. The LNCaP cells are harvested, and a cell suspension is prepared to be stained with Hoechst 33342, followed by washing and resuspension in 2% FBS in PBS. 2. The stained cells are analyzed with FACS and sorted in the cell cycle phases of G1, S and G2/M. Using the cytospin, the sorted cells are collected on the glass slide and visualized in the DAPI channel to assess the nuclei in different cell phases. 3. The sorted cells are seeded and incubated overnight on anti-PSA coated membranes, membranes are stained and visualized for PSA spots in the FITC channel. Created with biorender.com.

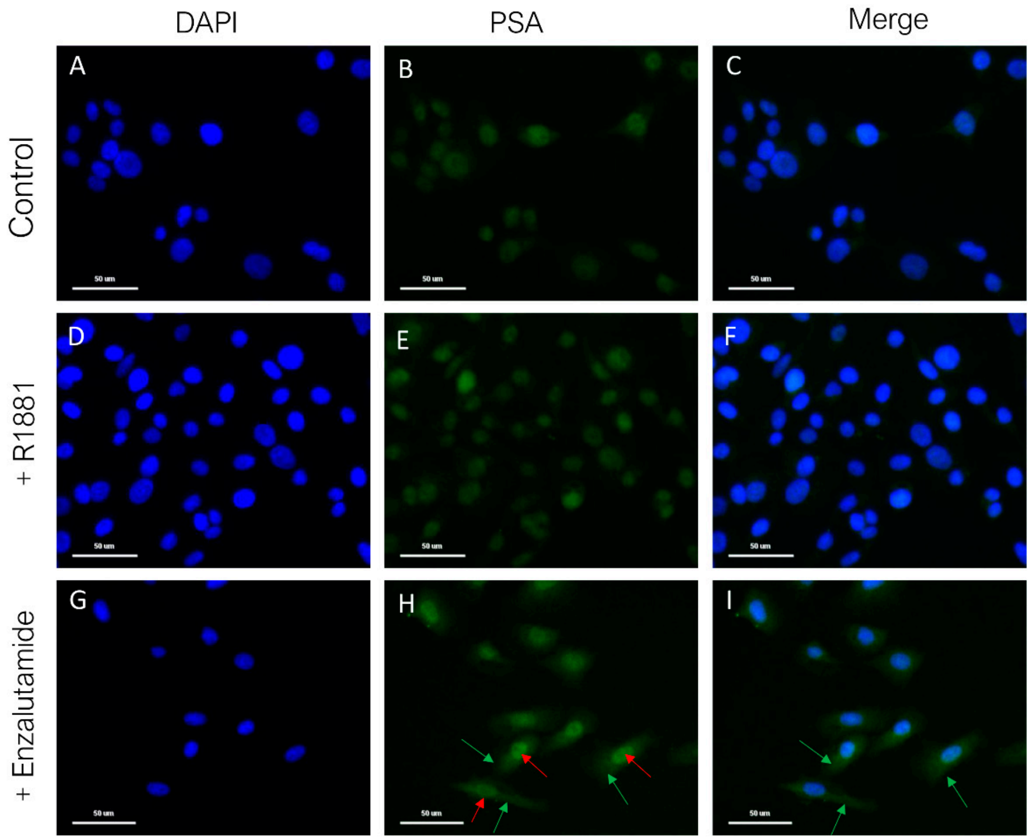

Figure S4: Immunofluorescence staining in PWR-1E cells. Blue represents nuclear stain (DAPI), green represents PSA (FITC). The nucleus staining is shown (in DAPI) for control, R1881 and Enzalutamide treated cells in (**A**), (**D**), and (**G**) respectively. The PSA staining is shown (in FITC) for control, R1881 and Enzalutamide treated cells in (**B**), (**E**), and (**H**) respectively. The overlay of all channels for the control, stimulated and inhibited condition is shown in (**C**), (**F**) and (**I**) respectively. PSA was observed to be low in control (**B**) and stimulated conditions (**E**) but upregulated in inhibited condition (**H**) as indicated in green arrows. Some non-specific staining of the nuclei was observed in the FITC channel as indicated with red arrows. Scale bar = 50 μm.

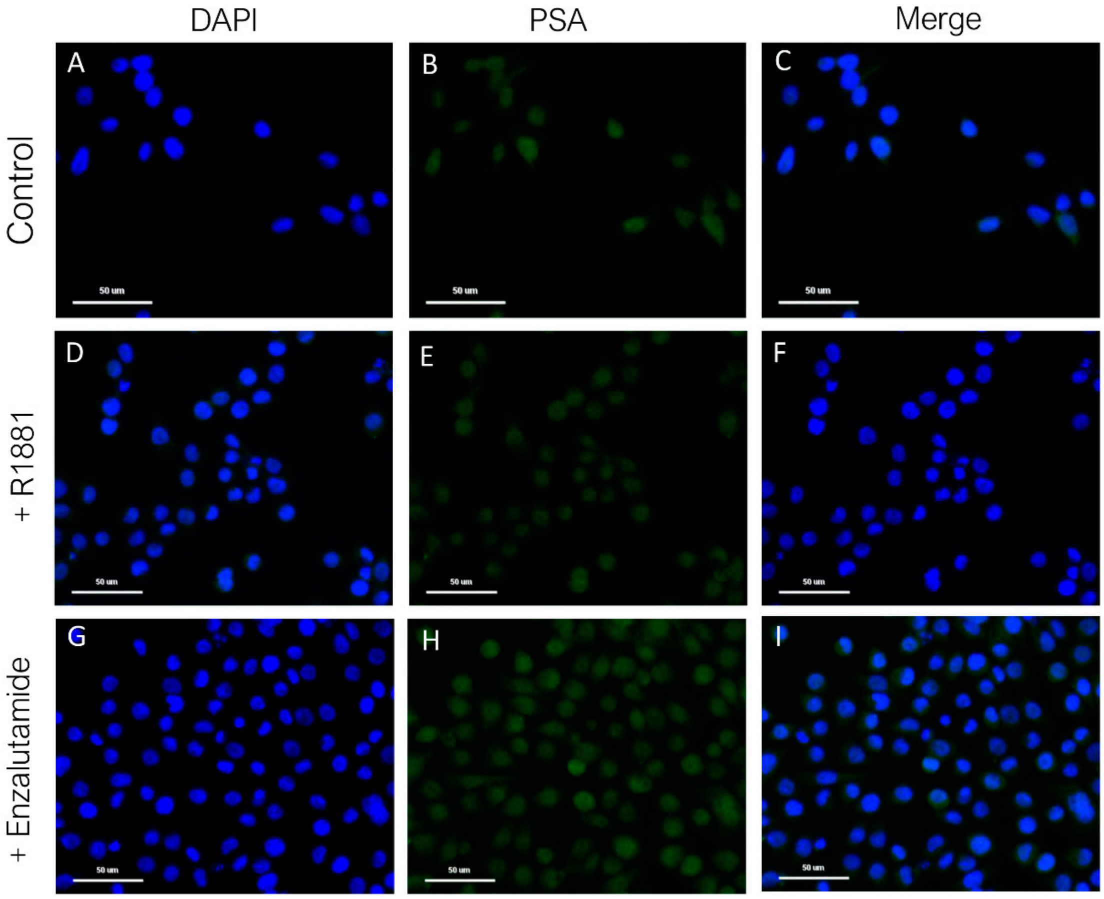

Figure S5: Immunofluorescence staining of RWPE-1 cells. Blue represents nuclear stain (DAPI), green represents PSA (FITC). The nucleus staining is shown (in DAPI) for control, R1881 and Enzalutamide treated cells in (**A**), (**D**), and (**G**) respectively. The PSA staining is shown (in FITC) for control, R1881 and Enzalutamide treated cells in (**B**), (**E**), and (**H**) respectively. The overlay of all channels for the control, stimulated and inhibited condition is shown in (**C**), (**F**) and (**I**) respectively. No PSA expression in the cytoplasm of the cell was observed. Scale bar = 50 μm.

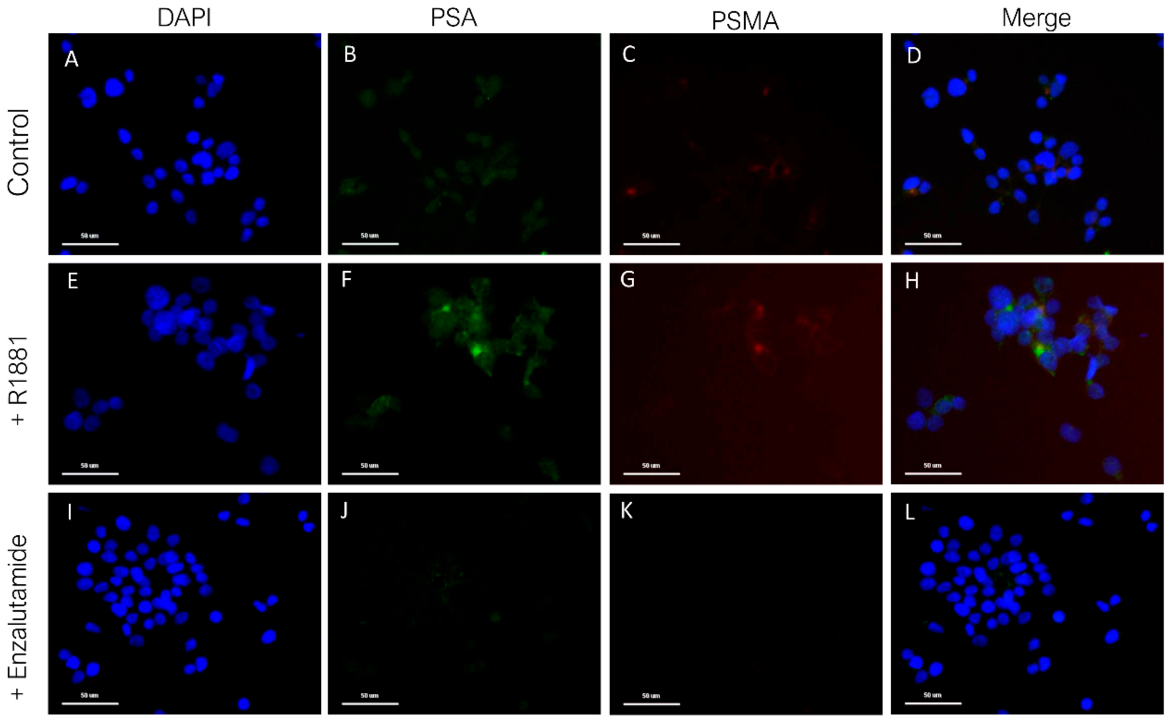

Figure S6: Immunofluorescence staining of 22Rv1 cells. Blue represents nuclear stain (DAPI), green represents PSA (FITC) and red represents PSMA (PE). The nuclear staining is shown (in DAPI) for control, R1881 and Enzalutamide treated cells in (**A**), (**E**), and (**I**) respectively. The PSA staining is shown (in FITC) for control, R1881 and Enzalutamide treated cells in (**B**), (**F**), and (**J**) respectively. The PSMA staining is shown (in PE) for control, R1881 and Enzalutamide treated cells in (**C**), (**G**), and (**K**) respectively. The overlay of all channels for the control, stimulated and inhibited condition is shown in (**D**), (**H**) and (**L**) respectively. The cells show the presence of PSA and PSMA in very low levels in the control state (**B**) and (**C**) respectively. The stimulation and inhibition show no effect on the expression levels of PSA in (**F**), (**J**) and PSMA in (**G**) and (**K**) respectively. Scale bar = 50 µm.

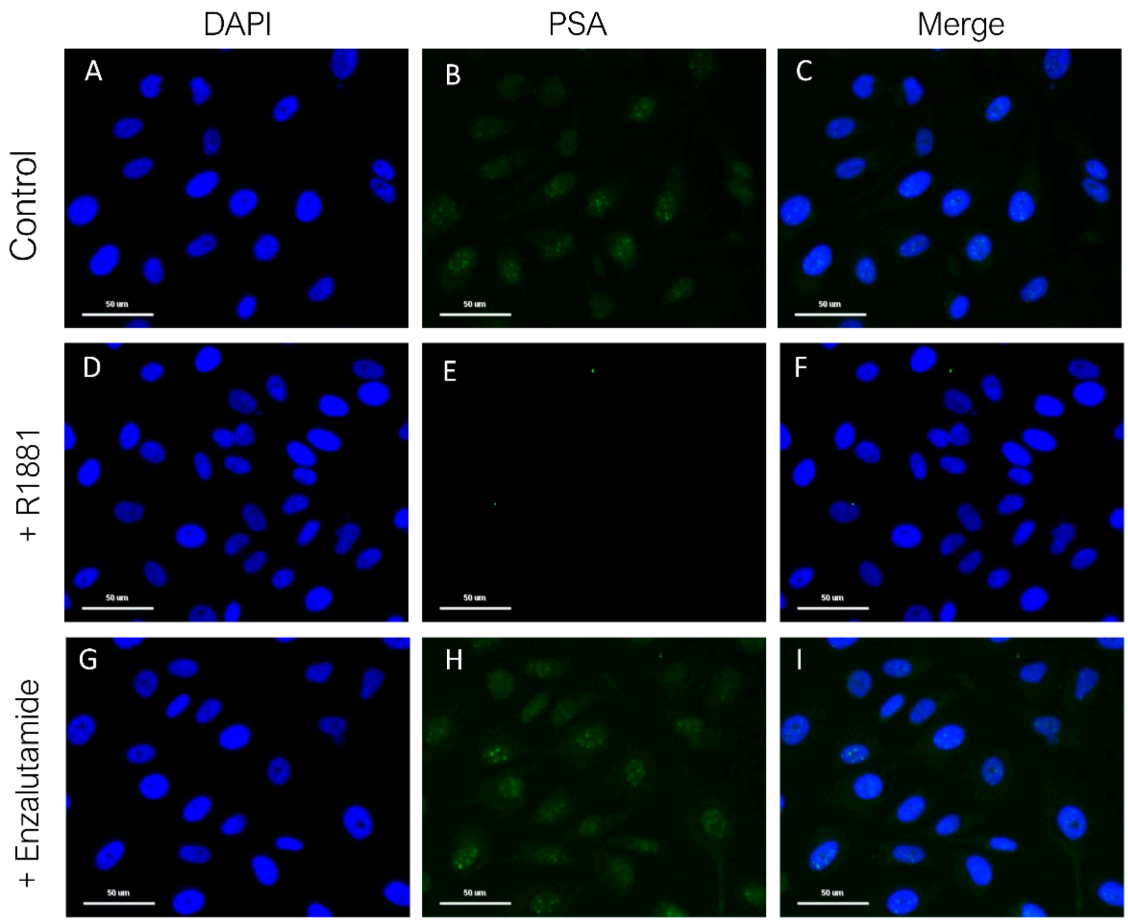

Figure S7: Immunofluorescence staining of PC3 cells. Blue represents nuclear stain (DAPI), green represents PSA (FITC). The nucleus staining is shown (in DAPI) for control, R1881 and Enzalutamide treated cells in (**A**), (**D**), and (**G**) respectively. The PSA staining is shown (in FITC) for control, R1881 and Enzalutamide treated cells in (**B**), (**E**), and (**H**) respectively. The overlay of all channels for the control, stimulated and inhibited condition is shown in (**C**), (**F**) and (**I**) respectively. The PC3 cell lines do not exhibit PSA in the untreated state in (**B**) and showed no change with stimulation or inhibition in (**E**) and (**H**) respectively. Scale bar = 50 μm.

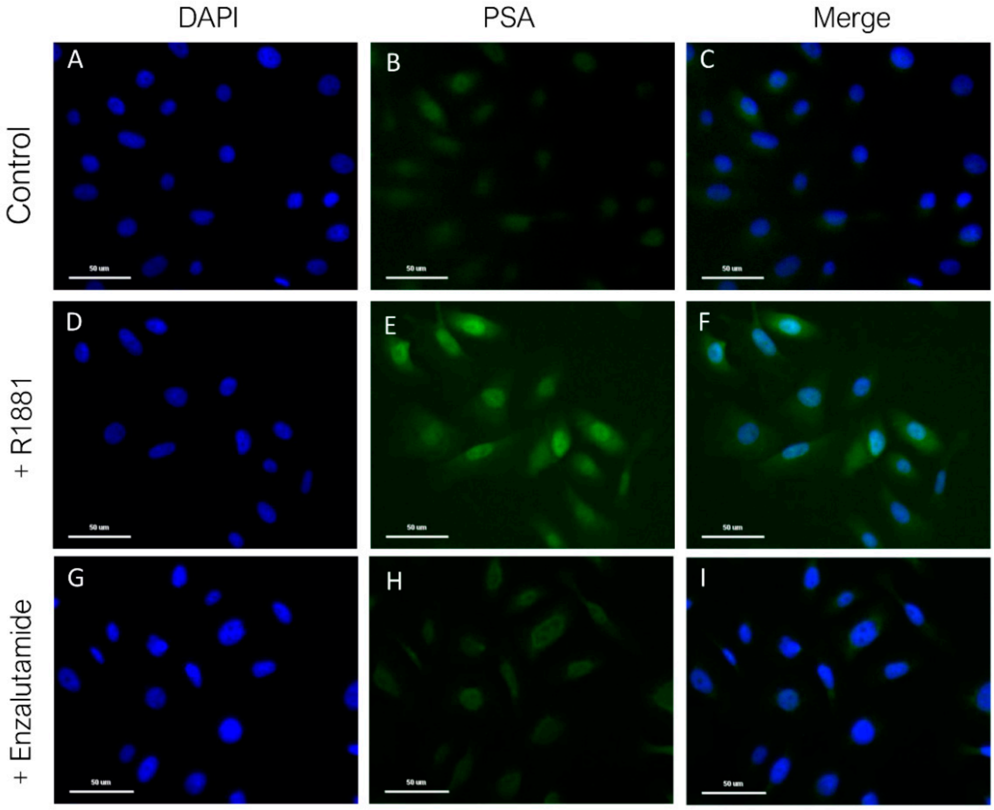

Figure S8: Immunofluorescence staining of RWPE-2 cells. Blue represents nuclear stain (DAPI), green represents PSA (FITC). The nucleus staining is shown (in DAPI) for control, R1881 and Enzalutamide treated cells in (**A**), (**D**), and (**G**) respectively. The PSA staining is shown (in FITC) for control, R1881 and Enzalutamide treated cells in (**B**), (**E**), and (**H**) respectively. The overlay of all channels for the control, stimulated and inhibited condition is shown in (**C**), (**F**) and (**I**) respectively. The cells of RWPE-2 show low expression of PSA in the untreated state in (**B**). With androgen stimulation, a small increase in PSA expression is observed as seen in (**E**), and subsequently decreased post-inhibition in (**H**) thereby, indicating a response to androgen therapy. Scale bar = 50 µm.

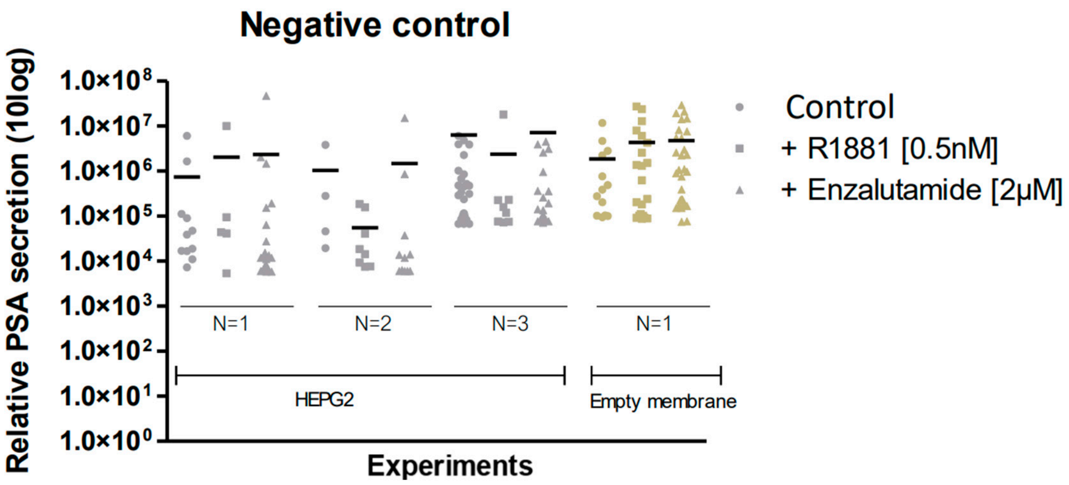

Figure S9: Relative intensities from membranes seeded with HepG2 cells (after 24 hours) and empty membranes. The mean value (represented as black dash) of all spots of the HepG2 cells is used as a threshold for the other cell lines All spots with a value of less than the mean value of HepG2 cells is considered as artifacts.

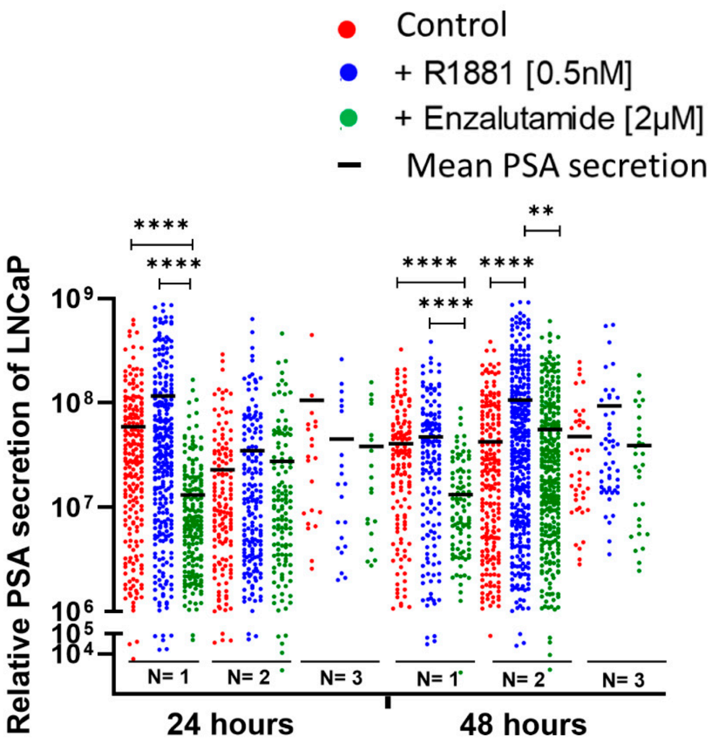

Figure S10: Scatter plot showing the effect of androgen treatment on PSA secretion from LNCaP cells.

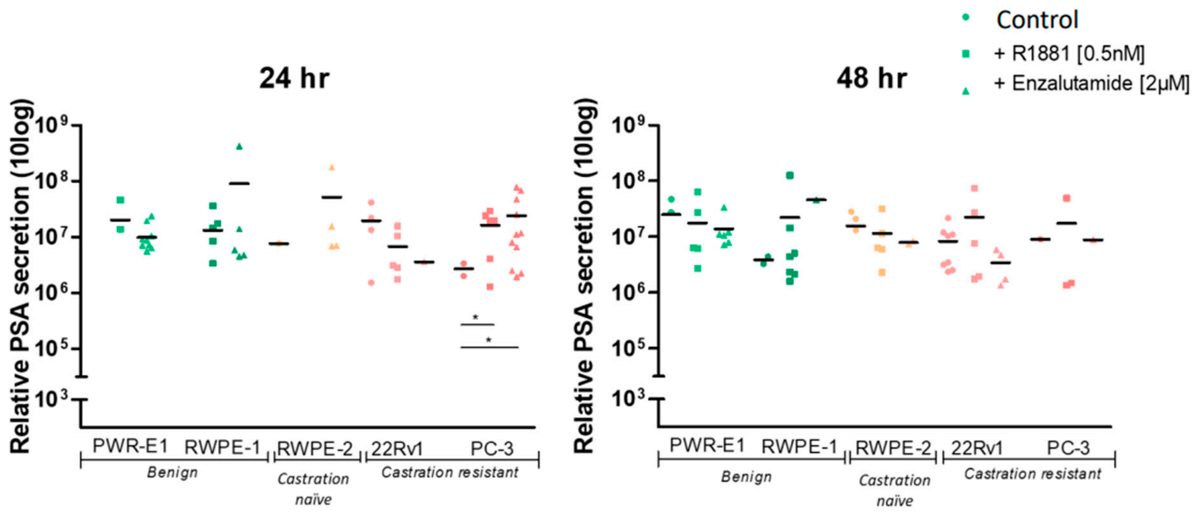

Figure S11: Single-cell PSA secretion from PWR-1E, RWPE-1, RWPE-2, 22Rv1 and PC3 (N = 3). Few spots were detected on the membranes due to which the effect of stimulation and inhibition on the PSA secretion could not be observed. Mean relative PSA secretion represented with black dash.

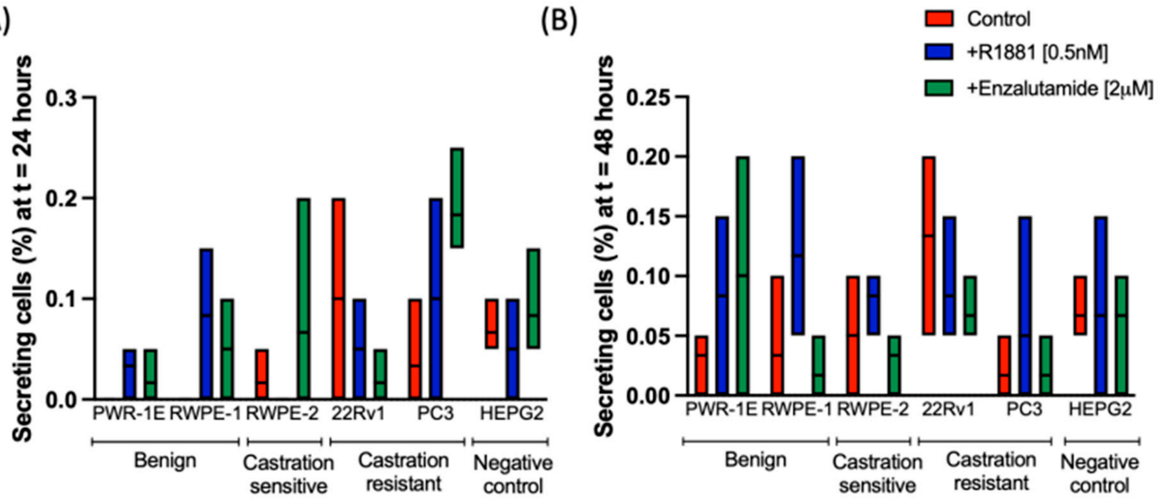

Figure S12: Percentages secreting cells of PWR-1E, RWPE-1, RWPE-2, 22Rv1, PC3 and HepG2 after (A) 24- and (B) 48-hour time points. The percentages of PSA secreting cells of the PCa cell lines are comparable to the negative control cell line HepG2.

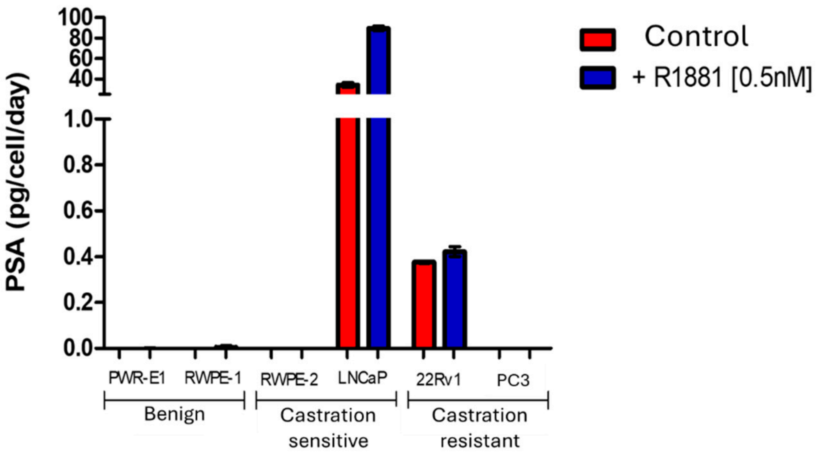

Figure S13: Effect of androgen treatment on bulk PSA from cell lines PWR-1E, RWPE-1, RWPE-2, LNCaP, 22Rv1 and PC3. ELISA performed for untreated (red) and R1881 treated (blue) cells. An increase in PSA secretion was observed in the LNCaP cell line upon androgen stimulation. PSA secretion is calculated in pg/cell/day.

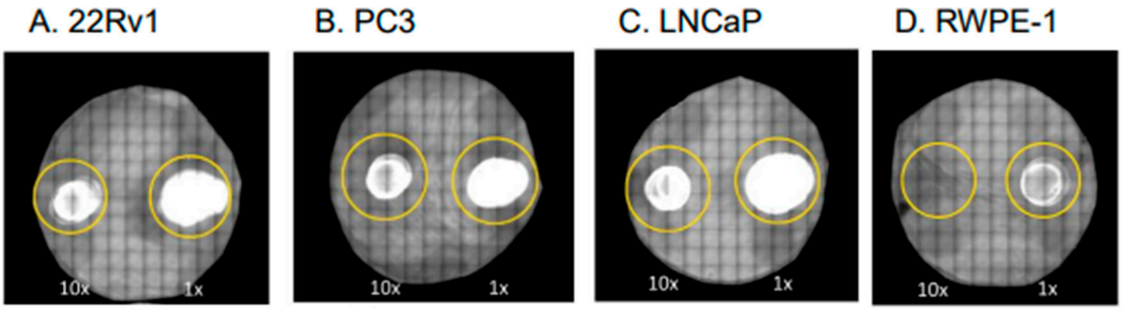

Figure S14: Dot blot to measure Progranulin from cell lines. The cell supernatant (2 μl) was spotted on the nitrocellulose membrane (1X) along with a sample diluted 10 times(10X). The presence of Progranulin was detected in higher intensities in the supernatant in all metastatic cell lines and HepG2, compared to RWPE-1 and GAPDH. The spotted area on the membranes is highlighted with yellow circles. Membranes imaged with Texas Red with exposure of 30ms.

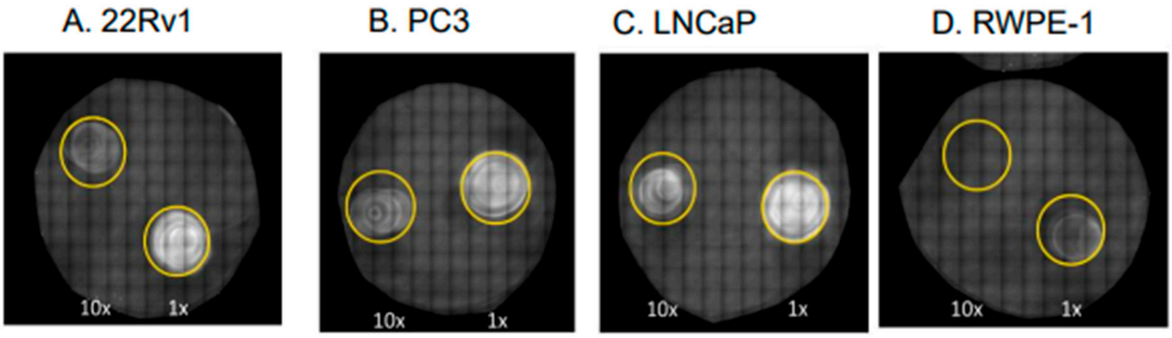

Figure S15: Dot blot to measure Cathepsin D from cell lines. The cell supernatant (2 μl) was spotted on the nitrocellulose membrane (1X) along with a sample diluted 10 times (10X). The presence of Cathepsin D was detected in high intensities in the supernatant in all metastatic cell lines and HepG2, compared to RWPE-1 and GAPDH. The spotted area on the membranes is highlighted with yellow circles. Membranes imaged with Texas Red with exposure of 30ms.
