# Supplementary material for: Secretome Analysis of Prostate Cancer Cell Lines Reveals Cell Cycle-Dependent PSA Secretion and Potential Biomarkers
Source: Cancers (Basel). 2025 Feb 20;17(5):721. doi: 10.3390/cancers17050721 (PMC11899065; doi:10.3390/cancers17050721)
Supplement: Supplementary file 1 [file cancers-17-00721-s001.zip › Supplementary_Tables.pdf]

Supplementary Tables:

Supplementary Table S1: Characteristics of PCa cell lines

Cell line (Cell Identifier)	Source	Metastasis	PSA secretion	Cellular PSA
**Benign**				
PWR-1E (RRID: CVCL_3775)	Epithelial	No	No	Yes
RWPE-1 (RRID: CVCL_3791)	Epithelial	No	No	Yes, when exposed to R1881
**Castration-sensitive**				
RWPE-2 (RRID: CVCL_3792)	Epithelial	No	-	Yes, when exposed to R1881
LNCaP (RRID: CVCL_0395)	Epithelial (lymph node)	Bone metastasis	Yes	Yes
**Castration-resistant**				
22Rv1 (RRID: CVCL_1045)	Epithelial	Bone metastasis	Yes	Yes
PC3 (RRID: CVCL_0035)	Epithelial (vertebral)	Bone metastasis	No	No

Supplementary Table S2: Antibodies used for immunofluorescence staining, single cell secretion staining, ELISA and dot blot assay.

Antibody	Working concentration	Catalog No.	Manufacturer
**Primary Antibodies**			
Rabbit Anti-PSA	2 μg/mL	ab19554	Abcam, Cambridge, UK
Mouse Anti-PSMA- PE (FOLH1)	1 μg/mL	342504	BioLegend, San Diego, USA
Mouse Anti-PSA	25 μg/mL	10-P21A	Fitzgerald Industries International, USA
PSA peptide	0 – 60 ng/mL	ab41421	Abcam, Cambridge, UK
Goat Anti-Progranulin	1 μg/mL	EVBIEB11324	Everest Biotech, USA
Rabbit Anti-STEAP2	1.2 μg/mL	20201-1-AP	Proteintech, UK
Rabbit Anti-Prostein	20 μg/mL	PA5-53915	Invitrogen, ThermoFischer Scientific, USA
Rabbit Anti-Cathepsin-D	14 μg/mL	PA5-17353	Invitrogen, ThermoFischer Scientific, USA
Rabbit Anti-GAPDH	0.42 μg/mL	2118S	Cell Signaling Technology, USA
Secondary Antibodies			
Goat Anti-rabbit Alexa fluor 488	2 μg/mL	ab150077	Abcam, Cambridge, UK

Goat Anti-rabbit-HRP	3 µg/mL	P044801-2	Dako, Agilent, Santa Clara, USA
Donkey Anti-rabbit Alexa fluor 594	20 µg /mL	**A-21207**	Invitrogen, ThermoFischer Scientific, USA
Donkey Anti-goat Alexa fluor 594	20 µg /mL	A-11058	Invitrogen, ThermoFischer Scientific, USA

Supplementary Table S3: HepG2 secretion and no cell secretion (A.U.) values from 24-hour timepoint.

	**HepG2**			**No cell**
	**N=1**	**N=2**	**N=3**	**(N=1)**
Minimum	$5.40 * 10^3$	$6.07 * 10^3$	$6.78 * 10^4$	$7.53 * 10^4$
Maximum	$4.77 * 10^7$	$1.53 * 10^7$	$1.56 * 10^8$	$2.97 * 10^7$
Median	$1.68 * 10^4$	$1.44 * 10^4$	$1.95 * 10^5$	7.61 * 105
Mean	$1.72 * 10^6$	$8.57 * 10^5$	$5.29 * 10^6$	$3.65 * 10^6$
Std. Deviation	$7.87 * 10^6$	$3.23 * 10^6$	$2.70 * 10^7$	$6.99 * 10^6$

Supplementary Table S4: Relative PSA secretion (A.U.) values from LNCaP cells for 24- and 48-hour time points.

	**PSA secretion (A.U) for 24 hours (N=3)**			**PSA secretion (A.U) for 48 hours (N=3)**		
	**Control**	**+R1881**	**Enzalutamide**	**Control**	**+R1881**	**Enzalutamide**
Minimum	$8.70 * 10^5$	$2.94 * 10^5$	$9.08 * 10^5$	$1.02 * 10^6$	$7.97 * 10^4$	$3.08 * 10^5$
Maximum	$6.23 * 10^8$	$4.55 * 10^9$	$2.37 * 10^8$	$3.85 * 10^8$	$1.52 * 10^9$	$1.17 * 10^9$
Median	$2.31 * 10^7$	$4.58 * 10^7$	$1.39 * 10^7$	$3.33 * 10^7$	$4.79 * 10^7$	$2.17 * 10^7$
Mean	$6.30 * 10^7$	$1.23 * 10^8$	$2.23 * 10^7$	$5.56 * 10^7$	$1.19 * 10^8$	$5.83 * 10^7$
Standard Deviation	$9.76 * 10^7$	$3.27 * 10^8$	$3.02 * 10^7$	$6.10 * 10^7$	$1.93 * 10^8$	$1.02 * 10^8$
25th percentile	$7.34 * 10^6$	$6.58 * 10^6$	$4.29 * 10^6$	$1.33 * 10^7$	$2.42 * 10^7$	$1.02 * 10^7$
75th percentile	$8.54 * 10^7$	$1.09 * 10^8$	$2.87 * 10^7$	$7.60 * 10^7$	$1.24 * 10^8$	$6.19 * 10^7$

Supplementary Table S5: Percentages of LNCaP cells secreting for 24- and 48-hour timepoints.

	**Cells (%) secreting for 24 hours**			**Cells (%) secreting for 48 hours**		
	**Control**	**+R1881**	**Enzalutamide**	**Control**	**+R1881**	**Enzalutamide**
Minimum	1.10	1.10	0.95	2.15	2.60	1.45
Maximum	11.55	16.45	11.15	11.50	18.70	17.00
Median	7.50	9.15	6.80	7.55	8.20	5.60
Mean	6.72	8.90	6.30	7.07	9.83	8.02
Standard Deviation	5.27	7.68	5.12	4.69	8.17	8.05
25th percentile	1.10	1.10	0.95	2.15	2.60	1.45
75th percentile	11.55	16.45	11.15	11.50	18.70	17.00

Supplementary Table S6: Total PSA secretion (A.U.) from LNCaP for 24- and 48-hour timepoints.

	**24 hours**			**48 hours**		
	**Control**	**+R1881**	**Enzalutamide**	**Control**	**+R1881**	**Enzalutamide**
Minimum	$2.45 * 10^{9}$	$1.14 * 10^{9}$	$8.47 * 10^{8}$	$2.30 * 10^{9}$	$5.19 * 10^{9}$	$1.33 * 10^{9}$
Maximum	$1.35 * 10^{10}$	$3.75 * 10^{10}$	$4.15 * 10^{9}$	$1.01 * 10^{10}$	$4.02 * 10^{10}$	$1.94 * 10^{10}$
Median	$3.99 * 10^{9}$	$6.83 * 10^{9}$	$3.22 * 10^{9}$	$6.37 * 10^{9}$	$7.90 * 10^{9}$	$1.64 * 10^{9}$
Mean	$6.65 * 10^{9}$	$1.52 * 10^{10}$	$2.74 * 10^{9}$	$6.24 * 10^{9}$	$1.78 * 10^{10}$	$7.45 * 10^{9}$
Standard Deviation	$5.98 * 10^{9}$	$1.96 * 10^{10}$	$1.70 * 10^{9}$	$3.88 * 10^{9}$	$1.95 * 10^{10}$	$1.03 * 10^{10}$
25th percentile	$2.45 * 10^{9}$	$1.14 * 10^{9}$	$8.47 * 10^{8}$	$2.30 * 10^{9}$	$5.19 * 10^{9}$	$1.33 * 10^{9}$
75th percentile	$1.35 * 10^{10}$	$3.75 * 10^{10}$	$4.15 * 10^{9}$	$1.01 * 10^{10}$	$4.02 * 10^{10}$	$1.94 * 10^{10}$

Supplementary Table S7: Relative mean PSA secretion values and percentage of secreting cells from PWR-1E, RWPE-1, RWPE-2, 22Rv1 and PC3 cell lines.

**Cell lines**	**Timepoint (hours)**	**Relative mean secretion (A.U.)**			**Secreting cells (%)**		
		Control	+R1881	+ Enzalutamide	Control	+R1881	+ Enzalutamide
**PWR-1E**	24	0	$2.00 * 10^{7}$	$4.00 * 10^{6}$	0	0.03	0.10
	48	$2.50 * 10^{7}$	$1.80 * 10^{7}$	$1.40 * 10^{7}$	0.03	0.01	0.02
**RWPE-1**	24	0	$1.30 * 10^{7}$	$9.10 * 10^{7}$	0	0.08	0.05
	48	$4.00 * 10^{6}$	$2.20 * 10^{7}$	$4.70 * 10^{7}$	0.03	0.10	0.02
**RWPE-2**	24	$8.00 * 10^{6}$	0	$5.20 * 10^{7}$	0.02	0	0.07
	48	$1.60 * 10^{7}$	$1.10 * 10^{7}$	$8.00 * 10^{6}$	0.05	0.08	0.03
**22Rv1**	24	$2.00 * 10^{7}$	$7.00 * 10^{6}$	$4.00 * 10^{6}$	0.10	0.05	0.02
	48	$8.00 * 10^{6}$	$2.20 * 10^{7}$	$3.00 * 10^{6}$	0.10	0.07	0.07
**PC3**	24	$3.00 * 10^{6}$	$1.60 * 10^{7}$	$2.40 * 10^{7}$	0.03	0.10	0.20
	48	$9.00 * 10^{6}$	$1.70 * 10^{7}$	$9.00 * 10^{6}$	0.02	0.05	0.02

Supplementary Table S8: PSA secretions (picogram/cell/day) from cell lines from ELISA.

	**PWR-1E**		**RWPE-1**		**RWPE-2**		**LNCaP**		**22Rv1**		**PC3**	
	**Control**	**+R1881**	**Control**	**+R1881**	**Control**	**+R1881**	**Control**	**+R1881**	**Control**	**+R1881**	**Control**	**+R1881**
Minimum	-0.002	0.000	-0.001	-0.004	-0.002	-0.003	32.300	87.940	0.373	0.407	-0.001	-0.002
Maximum	-0.001	0.001	-0.001	0.009	-0.001	0.000	35.570	90.910	0.378	0.437	0.000	-0.002
Median	-0.001	0.001	-0.001	0.003	-0.002	-0.002	33.940	89.430	0.375	0.422	-0.001	-0.002
Mean	-0.001	0.001	-0.001	0.003	-0.002	-0.002	33.940	89.430	0.375	0.422	-0.001	-0.002
Standard Deviation	0.001	0.001	0.000	0.009	0.001	0.002	2.308	2.103	0.004	0.021	0.000	0.000
25th percentile	-0.002	0.0001	-0.001	-0.003	-0.002	-0.003	32.30	87.940	0.3725	0.4069	-0.000	-0.002
75th percentile	-0.000	0.001	-0.000	0.009	-0.001	-0.000	35.570	90.910	0.378	0.436	-0.000	-0.001

Supplementary Table S9: Proteome Array pixel intensities (A.U.) of the proteins secreted by the cell lines.

		Pixel intensity of proteins (A.U)					
**Cell lines**	**Values (A.U)**	**Progranulin**	**IL-8**	**PSA**	**CathepsinD**	**Serpin**	**Enolase**
**LNCaP**	Minimum	2.91	0.22	11.82	1.77	0.21	0.29
	Maximum	3.02	0.24	12.93	2.33	0.96	0.30
	Mean	2.96	0.23	12.38	2.05	0.58	0.30
	Standard Error of Mean	0.05	0.01	0.55	0.28	0.38	0.01
	Median	2.96	0.23	12.38	2.05	0.58	0.30
	25th percentile	2.91	0.22	11.82	1.77	0.21	0.29
	75th percentile	3.02	0.24	12.93	2.33	0.96	0.30
**PC3**	Minimum	19.66	7.48	0.39	5.82	13.48	1.11
	Maximum	20.59	7.75	0.47	5.96	14.67	1.12
	Mean	20.12	7.62	0.43	5.89	14.08	1.12
	Standard Error of Mean	0.47	0.14	0.04	0.07	0.59	0.00
	Median	20.12	7.62	0.43	5.89	14.08	1.12
	25th percentile	19.66	7.48	0.39	5.82	13.48	1.11
	75th percentile	20.59	7.75	0.47	5.96	14.67	1.12
**22Rv1**	Minimum	10.85	0.19	2.21	6.44	2.98	4.48
	Maximum	11.61	0.38	2.30	6.80	3.78	5.24
	Mean	11.23	0.29	2.26	6.62	3.38	4.86
	Standard Error of Mean	0.38	0.10	0.05	0.18	0.40	0.38
	Median	11.23	0.29	2.26	6.62	3.38	4.86
	25th percentile	10.85	0.19	2.21	6.44	2.98	4.48
	75th percentile	11.61	0.38	2.30	6.80	3.78	5.24
